# Microbiome alterations in women with gestational diabetes mellitus and their offspring: A systematic review

**DOI:** 10.3389/fendo.2022.1060488

**Published:** 2022-12-08

**Authors:** Sara Farhat, Mahboobeh Hemmatabadi, Hanieh-Sadat Ejtahed, Nooshin Shirzad, Bagher Larijani

**Affiliations:** ^1^ School of Medicine, Tehran University of Medical Sciences, Tehran, Iran; ^2^ Scientific Research Center, Tehran University of Medical Sciences, Tehran, Iran; ^3^ Endocrine Research Center, Valiasr Hospital, Imam Khomeini Hospital Complex, Tehran University of Medical Sciences, Tehran, Iran; ^4^ Obesity and Eating Habits Research Center, Endocrinology and Metabolism Clinical Sciences Institute, Tehran University of Medical Sciences, Tehran, Iran; ^5^ Endocrinology and Metabolism Research Center, Endocrinology and Metabolism Clinical Sciences Institute, Tehran University of Medical Sciences, Tehran, Iran

**Keywords:** microbiota, microbiome, gestational diabetes mellitus, pregnancy-induced diabetes, offspring

## Abstract

**Aims:**

Gestational diabetes mellitus (GDM) is a metabolic disorder that might predispose pregnant women to develop type 2 Diabetes Mellitus or lead to severe adverse outcomes in their offspring. One of the factors that have been thought to be involved in the pathology behind this disorder is the microbiome. In this systematic review, we comprehensively review the documents regarding the microbiota alterations in different tracts of pregnant women with GDM and their offspring.

**Methods:**

A comprehensive search was conducted in major databases including MEDLINE (PubMed), Scopus, and Web of sciences up to August 2021. Data on the demographics, methodology, and microbiome alterations were extracted and classified according to the type of microbiome in pregnant women with GDM and their offspring. The quality of studies was assessed using the Newcastle-Ottawa Scale (NOS).

**Results:**

In 49 articles which were retrieved, the findings were variable on the level of changes in alpha and beta diversity, enrichment or depletion in phyla, genera, species and OTUs, in each microbiome type. Although there were some inconsistencies among the results, a pattern of significant alterations was seen in the gut, oral, vaginal microbiome of women with GDM and gut, oral, and placental microbiome of their offspring.

**Conclusion:**

Even though the alteration of the microbiome of the different tracts was seen in the cases of GDM, the inconsistency among the studies prevents us from identifying unique pattern. However, the results seem promising and further studies that overcome the confounding factors related to the demographics and methodology are needed.

## Introduction

Pregnancy is a complex phenomenon in which different metabolic, hormonal, and physiological changes occur in the woman’s body, ensuring the optimum environment for fetal development and subsequently causing various responses in the female body. One of these changes is the transient state of hyperglycemia that usually develops during pregnancy (1). The increase in the pro-inflammatory cytokines and metabolic hormones while the pregnancy progresses, mostly during the third trimester, induces a decrease in insulin sensitivity. In some cases, this might lead to the development of a pathological state of glucose intolerance during pregnancy, known as Gestational Diabetes Mellitus (GDM) (1).

As it forms a great burden on the health care system with a significant prevalence of approximately 5-20% of pregnancies, there have been some attempts to modify the risk factors that might lead to GDM, by decreasing weight, diet control, and medical nutrition therapy. Medical treatment with insulin or hypoglycemic drugs might be needed in case of the development of GDM (2). Recently, special attention has been given to assessing the role of microbiota in the occurrence of the disease to achieve the best therapeutic plans (3, 4).

Microbiota is the collection of microorganisms that reside in the mucosal surfaces and the skin in a symbiotic interaction. The gut, for instance, hosts an estimated number of 100 trillion bacteria, archaea, viruses, and eukaryotic microbes that colonize mainly the distal colon (5). The human vaginal, oral, respiratory and uterine microbiota are major components of the mucosal surfaces as well (6). The human microbiome is believed to contribute to different physiological and pathological mechanisms. Immunomodulation of the host, defense against pathogenic bacteria by preventing their attachment to mucosal surfaces, digestion, metabolism, production and extraction of nutrients and vitamins to be absorbed by epithelial cells, are some of the proposed functions of the human gut microbiome (1, 6). Studies have concluded a relation between human microbiome and different pathologies such as metabolic syndrome and its components including obesity, hyperglycemia, and insulin resistance (7, 8). Various interpretations were proposed for the role of microbiota in the mechanisms behind these pathologies. For instance, it is believed that microbiota induces obesity through fermentation of dietary fibers and inducing an overproduction of short-chain fatty acids (SCFAs). It can also lead to the development of type 2 diabetes mellitus (T2DM) by increasing membrane transport of sugars and branched chain amino acids and oxidative stress response (9). Additionally, an altered microbiome is thought to induce insulin resistance through a low-grade inflammation mediated by lipopolysaccharides (LPS) pathways that are markedly increased in patients who consume a high-fat diet (1).

Special attention was paid recently to study the connection between the female’s body microbial communities with the development of GDM. It has been noticed that the microbiome undergoes some alterations both at the time of pregnancy and postpartum, particularly in the gastrointestinal, oral, and vaginal tracts (10, 11).

However, apparently, not only the mother’s microbiome is affected, since the dysbiosis was also seen in the microbiome of the neonates of pregnant women with GDM in their intestinal tracts and oral cavities. A specific microbial profile was witnessed in their placenta as well (12–14).

This correlation ignites many questions regarding the association between GDM and microbiota, and whether there is enough evidence to develop management strategies to prevent or treat GDM using the human microbiome modulating approaches. This systematic review aims to determine the current evidence regarding the association of the maternal and neonatal microbiota composition with gestational diabetes mellitus and to discuss the possibility of the management of GDM using the microbiome.

## Methods

We conducted a systematic review in accordance with the Preferred Reporting Items for Systematic Reviews and Meta-Analyses (PRISMA) statement.

### Search strategy

MEDLINE/PubMed, Scopus, and Web of Science databases have been searched using free texts and MeSH terms in titles and abstracts, including studies published till august 2021. The reference list of relevant reviews was screened manually.

The key words used in the search strategies were as follows: ((“Microbiota”[Mesh]) OR “Gastrointestinal Microbiome”[Mesh] OR Microbiota[tiab] OR microbiome[tiab] OR microbiomes[tiab] OR “microbial profile”[tiab] OR microflora[tiab] OR microfloras[tiab] OR flora[tiab] OR “microbial community”[tiab] OR “microbial communities”[tiab] OR “gut microbial composition”[tiab] OR “faecal microbial composition”[tiab] OR “intestinal microbial composition”[tiab]) AND (“Diabetes, Gestational”[Mesh] OR “gestational diabetes mellitus”[tiab] OR “gestational diabetes”[tiab] OR “Diabetes, Pregnancy-Induced”[tiab] OR “Pregnancy-Induced Diabetes”[tiab] OR “Pregnancy Induced Diabetes”[tiab] OR “Diabetes Mellitus, Gestational”[tiab]).

After removing the duplicates of the obtained articles, the abstracts of the articles were screened based on the inclusion and exclusion criteria.

### Inclusion and exclusion criteria

The articles were included in the study if they (1): were observational studies including cohort or prospective, cross-sectional, and case-control studies, (2) investigated the microbiota in pregnant women with GDM and/or their offspring as participants, (3) were conducted on humans, (4) were published in English language.

The articles were excluded if they were reviews and systematic reviews, animal experiments, clinical trials, protocols, conference papers, case reports and letters to the editor, in addition to articles that are irrelevant to the research topic. The outcomes of interest that will be compared between the different participants are:

The oral, vaginal and gut microbiome changes in pregnant women who developed GDM, as well as the oral, placental and gut microbiome changes in their offspring.

### Data extraction and quality assessment

The following data items will be recorded: first author, study year, study type and setting, country in which study was conducted, time of analysis, ethnicity of participants, recruitment sample and its size, sample site, the type of analyzed microbiome and the technique used, criteria used to determine the presence of GDM in mothers, and the increase or decrease in microbiota profile (phylum, order, family, genus) in comparison to the healthy control group.

The quality of the studies was assessed taking the risk of bias into consideration using the New castle Ottawa Scale (NOS) checklist that contains three parameters of quality: (1) Selection of population, (2) comparability of groups, (3) exposure and outcome in case-control and cohort studies (15). Each study was assigned a score from 0 to 9. The NOS adapted for cross sectional studies was used for the quality assessment of the cross sectional studies. Each study was assigned a score from 0 to 10. Studies with scores equal to or above 7 were considered as high quality articles.

## Results

**Figure 1:**
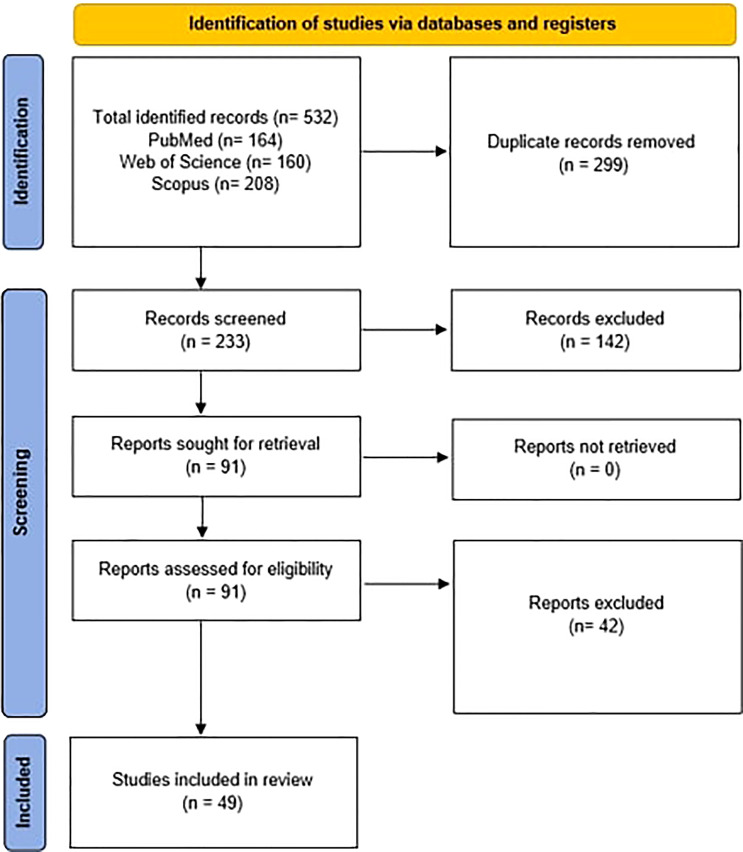
PRISMA flow diagram.

After searching the databases, a total of 532 articles were obtained. 164 articles were obtained from PubMed, 160 from Web of Science, and 208 from Scopus. After removing the duplicates, 233 remained for the initial screening of the title and abstract, from which 142 were excluded for not meeting the inclusion criteria and the remaining 91 were further assessed. Then 42 of these articles were excluded for not meeting the inclusion criteria after whole-paper examination, and therefore 49 articles were included for data extraction as summarized in the PRISMA flowchart (**Figure 1**).

### Study characteristics

The 49 included studies were classified for data extraction according to the type of the studied microbiome and were included in the **Supplementary Tables 1–7**. In some studies, the microbiome of more than one organ or microbiome of both women and their offspring were included, and the data of these articles was mentioned separately in each corresponding table, hence some articles might be listed in more than one table (16–24). The microbiome types were classified into the following categories: gut (30 articles), oral (8 articles), and vaginal (5 articles) microbiome of women with GDM in **Supplementary Tables 1, 2 and 3**, respectively; and gut (9 articles), oral (3 articles), and placental (4 articles) microbiome of offspring to women with GDM in **Supplementary Tables 4, 5 and 6**, respectively. The postpartum changes in microbiome of women with GDM were listed in **Supplementary Table 7**.

Most of the studies (28 out of 49) were conducted in China. Other countries included Denmark (19, 20, 25), Finland (17, 26, 27), Italy (28–31), United States of America (32–34), Australia (35), Brazil (21), Germany (36), India (37), Israel (16), Thailand (24), Malaysia (38), and Spain (39). The samples were analyzed at first, second, or third trimester or postpartum in the women with GDM, and in one study the sample was collected 1-2 days before delivery (18).

The timing of the offspring’s microbiome analysis was variable among the studies in a period extending from the first few hours after birth till 2 weeks. One study assessed the offspring microbiome for an even later time at 4 years (17).The diagnosis of GDM was determined according to the International Association of the Diabetes and Pregnancy Study Groups IADPSG criteria in most of the studies. In some studies, other criteria were used such as Chinese diagnostic criteria (40), Guidelines for the Prevention and Treatment of Type 2 Diabetes in China (41), International criteria (29, 31), World Health Organization criteria (21, 22, 42), American Diabetes Association (ADA criteria) (43–45), Hospital diagnostic criteria (46), National Diabetes Data Group (NDDG) criteria (24), Carpenter and Coustan criteria (32), American College of Obstetricians and Gynecologists (ACOG) criteria (47), and Malaysian Clinical Practice Guidelines (CPG) (38).

Criteria for inclusion and exclusion of the women and their offspring, although not identical, were mostly similar between the groups in the past medical history and medications. In many of the included studies in our review, women were excluded if they had used antibiotics in the last 2 weeks till 3 months prior to the first visit, had a history of chronic diseases and comorbidities such as pre-existing diabetes mellitus, hypertension, kidney, liver, gastrointestinal diseases and infections, and consumed probiotics prior to their entry in the study, in addition to other criteria detailed in the **Supplementary Tables 1–7**. However, in some studies the exclusion criteria were not determined. Body Mass Index (BMI) and age were determined in the inclusion criteria in some studies, but the exact limit was variable: the age ranged from 18-45 years with the minimal required age being at least 18 (22, 28, 35, 43), and BMI was variable between >25 (26, 27, 35), >25 but <30 (47), or ≤28 (48).

The samples collected for the gut microbiome analysis in the women with GDM included fecal samples in all the studies included as shown in **Supplementary Table 1**, while different sources of samples were collected for the assessment of their oral microbiome, such as oral swabs (16), saliva samples (18, 20, 22), supragingival plaque (45), saliva and dental plaque samples (49), and saliva, supragingival and subgingival plaques (23).

As for the vaginal microbiome, the samples were collected from vaginal mucosal swabs (16), posterior fornix (18), upper third of vagina (23), cervix (21), and vaginal secretions (30).

The offspring’s gut microbiome was analyzed based on the fecal stools samples in older offspring in few studies (17, 19, 31, 32), and on the meconium sample in the remaining studies as listed in **Supplementary Table 4**. Regarding the oral microbiome of the offspring, the samples included oral swabs except for one study that included saliva and pharyngeal aspirates (18). The placental microbiome of the offspring was studied from placental samples.

All of the studies used the 16S rRNA sequencing method to determine the composition of the microbiome.

### Gut microbiome changes in women with GDM

30 articles were suitable to be included in this category as shown in the **Supplementary Table 1**. Although the timing of analysis in each study was variable at both early and late stages of pregnancy, a specific pattern of microbiome alterations was witnessed among the different groups of women who developed GDM during pregnancy when compared to the women who did not develop it in the control groups.

Significant changes in the phyla Firmicutes, Bacteroidetes, Actinobacteria, Proteobacteria, Verrucomicrobia, and Fusobacteria have been recorded in the gut microbiome of mothers with GDM.

As for the Firmicutes, an increase in their frequency was seen in some studies (21, 29, 48, 50), sometimes manifested as an increase in the Firmicutes/Bacteroidetes ratio (24, 26, 50). However, Firmicutes were observed to be decreased in other studies (23, 51, 52), and in two other studies, the abundancy of Firmicutes was not significantly different between the two compared groups (28, 35). Totally, the phylum Firmicutes showed a significant alteration in the gut microbiome of pregnant women with GDM, with mostly being increased.

This inconsistency was also seen in the phylum Bacteroidetes, as it was increased in most of the studies (23, 46, 51–53), while it was not different among the compared groups in two studies (28, 35) and it was decreased in one study (29). The phylum Proteobacteria showed a variable change in its frequency in the gut microbiome of the pregnant women with GDM (51).

Actinobacteria phylum was increased according to Hou et al. (51), but its expression was otherwise decreased in few other studies (29, 52, 53). Verrucomicrobia was mostly increased (51, 54), and Fusobacteria was increased in Hou et al.’s study (51).

Overall, and as it is shown in **Table 1**, the phyla Bacteroidetes, Firmicutes, and Verrucomicrobia were increased in the gut microbiome of women with GDM, while the expression of the phylum Actinobacteria was reduced.

**Table 1:** Summary of the gut microbiome alterations in offspring to patients with GDM (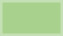
= Increased, 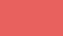
= Decreased, 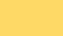
= Variable).

Microbiome	Phylum	Class	Order	Family	Genus	Species
**Gut microbiome of women with GDM**						
							**Bacteroidetes Firmicutes Verrucomicrobia**	***Gammaproteobacteria***	***Actinomycetales*** ***Coriobacteriales***	***Coriobacteriacea*** ***Enterobacteriacea*** ***Leuconostocaceae*** ***Micrococcaceae*** ***Ruminococcaceae***	***Actinomyces*** ***Bacteroides*** ***Blautia*** ***Desulfovibrio*** ***Lachnospiraceae*** ***Leuconostoc*** ***Parabacteroides*** ***Prevotella***	***Bacteroides caccae*** ***Bacteroides massiliensis*** ***Bacteroides thetaiotaomicron*** ***Catenibacterium mitsuokai*** ***Citrobacter*** ***Clostridium colinum*** ***Coprococcus comes*** ***Klebsiella variicola*** ***Parabacteroides distasonis*** ***Ruminococcus bromii*** ***Streptococcus infantis***
	**Actinobacteria**	**-**	**-**	**-**	***Bifidobacterium***	***Bacteroides vulgatus*** ***Lactobacillus rogosae*** ***Prevotella copri***
	**Proteobacteria**	**-**	**-**	**-**	***Collinsella*** ***Eubacterium*** ***Lactobacillus*** ***Rothia***	**-**

Genera including *Actinomyces* (19, 54), *Bacteroides* (28, 40, 44, 52–54), *Blautia* (19, 29, 55, 56), and *Prevotella* (21, 54) were mostly enriched in the gut microbiome of the women with GDM, while some other genera were depleted such as *Bifidobacterium* (40, 41, 55, 57).

It should be noted, however, that some of these studies were not qualified as high-quality according to the NOS checklist (21, 28, 41, 44, 46, 50, 51, 56), which might affect the efficacy of these results.

### Oral microbiome changes in women with GDM

The oral microbiome of women with GDM was assessed in 7 studies as well, and a pattern of alteration was observed as shown in **Supplementary Table 2**.

The phylum Firmicutes was decreased according to Wang et al. (18). However, the pattern of alteration was not as well established as it was for the gut microbiome of women with GDM, since in one study only alpha diversity was decreased without changes in beta diversity (22), and in two other studies no differences were seen in the phyla and genera (21, 49). These 2 studies, however, were not considered as high-quality studies according to the NOS checklist. In the remaining studies, a broad spectrum of changes in the families and genera was observed, such as an increase in *tuberculosis bacilli*, Black-pigmented bacteria, *Capnocytophaga*, *actinomycetes*, *Christensenellaceae, Ruminococcaceae* and *Enterobacteriaceae* and other genera (23, 45) and a decrease in others such as oral *streptococci, lactobacilli* (45), and *Bifidobacterium* (22).

Although not very specific, this pattern of alteration demonstrates a correlation between GDM and the composition of the oral microbiota.

### Vaginal microbiome changes in women with GDM

5 studies assessed the vaginal microbiome of women with GDM and their results appear in **Supplementary Table 3**. While the number of articles might not be sufficient to draw a conclusion, it still reflects the fact that the vaginal microbiome is affected by the status of GDM in the mother.

According to Zhang et al. (58), *Lactobacillus listeri, Lactobacillus amylovorus*, and *Lactobacillus fructivorans* were associated with the presence of GDM, while constitutional ratio of *vaginal L inersclone* was decreased.

In the research of Cortez et al. (21), the phyla Firmicutes and Proteobacteria were both increased but the change was not statistically significant. Moreover, Di Paola et al. (30) demonstrated a higher alpha diversity in the GDM group. Contrarily, Wang et al. did not detect any variation in the vaginal microbiome among the two groups (18).

Additionally, changes in microbial composition for the oral, vaginal, and rectal compartments in the women with gestational diabetes when compared with healthy pregnancies were seen in a study conducted by Solt et al., manifested by a skewed prevalence for multiple genera (16).

### Gut microbiome changes in offspring of women with GDM

As it was mentined in the microbiome of the women with GDM, alterations of the offspring gut microbiome was seen in some studies as well, from which 9 articles are included in our systematic review as shown in **Supplementary Table 4**. The phylum Proteobacteria was increased in the offspring gut in two articles (31, 59), while it was decreased in two others (33, 42). As for Bacteroidetes, it was seen to be both decreased (59) and increased (31, 33), and the Genus *Prevotella* and parent family *Prevotellaceae* that belong to this phylum were decreased according to Crusell et al. and Su et al. (25, 59). The frequency of Firmicutes was also assessed where it was found to be decreased in Hu et al. (33), increased in Chen et al. (42) and Crusell et al.’s study (25). Regarding Actinobacteria, its expression was increased in 2 studies (31, 59). Furthermore, Koren et al. demonstrated an increased beta-diversity in infants of age 1 month and 6 months, but by 4 years of age, children had a beta-diversity similar to mothers at T1 (at 13.84 ± 0.16 weeks of gestation) (17). Some genera correlated with GDM in another study such as *Blautia, Coprococcus, Roseburia* and *Sutterella* (18), and a decreased expression of *Lactobacillus, Flavonifractor, Erysipelotrichaceae* and unspecified families in *Gammaproteobacteria* was reported in Soderborg et al. (32). However, Sililas et al. have not observed any alterations in the meconium of the offspring (24).

All these studies were qualified to be of high-quality, except for 1 study (33) that was not qualified according to the New Castle Ottawa Scale.

### Oral microbiome changes in offspring of women with GDM

According to our review, there were only few studies that assessed the oral microbiome of the offspring to women with GDM, but alteration of the microbiome was seen in all of them, as manifested in **Supplementary Table 5**. For instance, consistency of microbial variation across mothers with GDM and their neonates in bacterial abundance was seen according to Wang et al. (18). Moreover, in the study of He et al. (60), higher alpha diversity was witnessed in the group of offspring to women with GDM especially in the Bacteroidetes phylum.

Furthermore, some genera were specific for this group as well as it was described in the study of Singh et al. (37), including *Alistipes, Streptococcus, Faecalibacterium, Prevotella, Bacteroidetes, Bifidobacterium, Corynebacterium, Ureaplasma* and *Weissella.* Nevertheless, it should be taken into consideration that two of these studies were not qualified to be of high-quality according to the NOS checklist (37, 60).

### Placental microbiome changes in offspring of women with GDM

We found 4 studies based on our search that evaluated the placental microbiome alterations of the offspring (**Supplementary Table 6**), one of which could not assess the samples due to an inaccurate detection (47). A distinguished pattern of microbiome in the group of offspring to women with GDM,where more operational taxonomic units in GDM group was seen in Tang et al., although this study was not considered to have a high-quality according to the NOS checklist (61). Additionally, and based on the study of Zheng et al., Proteobacteria was increased while the other phyla including Bacteroidetes, Actinobacteria, Firmicutes were decreased in placental microbiome (62).

In the study of Bassols et al. (39), some families were enriched including *Coriobacteriaceae*, *Lachnospiraceae* and *Bradyrhizobiaceae* from the phyla Actinobacteria, Firmicutes and Proteobacteria phyla, respectively.

### Post-partum microbiome changes in women with GDM

Few studies evaluated the postpartum gut microbiome of women who were diagnosed with GDM during pregnancy. We summarized their findings in **Supplementary Table 7**. Although there is heterogeneity among these studies regarding the exact timing in which the study was conducted, a clear changing in the gut microbiome was observed.

For instance, Koren et al. who assessed the microbiome 1 month postpartum noticed a persisted high levels of between-individual variation in community composition that was observed in the third trimester (T3), where a relevant abundance of the genus *Streptococcus* was seen (17).

Fugmann et al. who assessed the gut microbiome at 3-16 months after delivery concluded that alpha diversity was similar between the two groups and no significant differences on lower taxonomic levels were observed, but a decreased abundance of the phylum Firmicutes and a an enriched expression of the family *Prevotellaceae* was detected (36).

2 studies that were conducted by Crusell et al. described an altered pattern of the gut microbiome 8 and 9 months postpartum (19, 25). They found that some OTUs were enriched in women with previous GDM including two *Faecalibacterium* OTUs, while some were decreased including *Faecalibacterium, Bacteroides* and *Isobaculum* (19). Likewise, in the other study some OTUs were increased such as *Bacteriodales* and *Treponema* while others including *Leptotrichia, Streptococcus, Neisseria, Weeksellaceae*, and *Atopobium* were decreased (25). Recently, Hasain et al. also demonstrated an increase in the phylum Bacteroidetes and a decrease in Firmicutes between 3 and 6 months postpartum (38).

A summary of all the microbiome changes in both women and their offspring in the phylum, class, gender, order, family, genus, and species is presented in the **Tables 1**–**6**.

**Table 2:** Summary of the gut microbiome alterations in offspring to patients with GDM (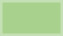
= Increased, 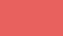
= Decreased, 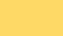
= Variable).

Microbiome	Phylum	Class	Order	Family	Genus	Species
**Gut microbiome of offspring to women with GDM**						
							**Actinobacteria** **Proteobacteria**	**-**	**-**	***Carnobacteriaceae*** ***Lachnospiraceae*** ***Streptococcaceae***	***Clostridium sensustricto*** ***Escherichia*** ***Parabacteroides*** ***Phascolarctobacterium***	
	**-**	**-**	**-**	***Prevotellaceae*** ***Micrococcaceae***	***Ethanoligenes*** ***Lactobacillus*** ***Megasphaera*** ***Prevotella*** ***Subdoligranulum*** ***Veillonella***	**-**
	**Bacteroidetes** **Firmicutes**	**-**	**-**	**-**	***Rothia***	**-**

**Table 3:** Summary of the oral microbiome alterations in patients with GDM (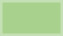
= Increased, 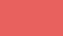
= Decreased, 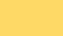
= Variable).

Microbiome	Phylum	Class	Order	Family	Genus	Species
**Oral microbiome of women with GDM**						
							**-**	**-**	**-**	***Enterobacteriaceae*** ***Christensenellaceae*** ***Ruminococcaceae*** ***Veillonellaceae***	***Actinomycetes*** ***Capnocytophaga*** ***Leptotrichia*** ***Prevotella***	**-**
	**Firmicutes**	**-**	**-**	**-**	**oral *streptococci*** ***Bifidobacterium*** ***Lactobacilli***	**-**
	**Proteobacteria**	**-**	**-**	**-**	**-**	**-**

**Table 4:** Summary of the oral microbiome alterations in offspring to patients with GDM (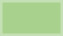
= Increased, 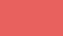
= Decreased, 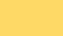
= Variable).

Microbiome	Phylum	Class	Order	Family	Genus	Species
**Oral microbiome of offspring to women with GDM**	**Bacteroidetes**	***Bacteroidia*** ***Clostridia***	***-***	***-***	***Alistpes*** ***Bacteroidetes*** ***Bifidobacterium*** ***Faecalibacterium*** ***Prevotella*** ***Streptococcus***	**-**

**Table 5:** Summary of the vaginal microbiome alterations in patients with GDM (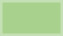
= Increased, 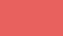
= Decreased, 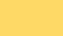
= Variable).

Microbiome	Phylum	Class	Order	Family	Genus	Species
**Vaginal microbiome of women with GDM**	**Firmicutes** **Proteobacteria** **(no statistical significance)**	**-**	**-**	***Aerococcaceae*** ***Enterobacteriaceae*** ***Lachnospiraceae*** ***Sutterellaceae***	***Bacteroides Veillonella*** ***Brevibacterium*** ***Enterococcus*** ***Fusobacterium*** ***Lactobacillus (predominant in both groups)*** ***Mobiluncus*** ***Prevotella***	***Lactobacillus amylovorus*** ***Lactobacillus fructivorans*** ***Lactobacillus listeri*** ***L acidophilus*** ***L crispatus*** ***L inersclone***

**Table 6:** Summary of the placental microbiome alterations in offspring to patients with GDM (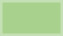
= Increased, 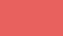
= Decreased, 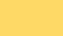
= Variable).

Microbiome	Phylum	Class	Order	Family	Genus	Species
**Placental microbiome of offspring to women with GDM**						
							**Proteobacteria**	**-**	***Coriobacteriales***	***Bradyrhizobiaceae*** ***Coriobacteriaceae*** ***Lachnospiraceae***	***Coprococcus*** ***Paraprevotella*** ***Ruminococcus***	**-**
	**-**	**-**	**-**	**-**	***Dyella*** ***Sulfuricella*** ***Veillonella***	**-**
	**Actinobacteria** **Bacteroidetes** **Firmicutes**	**-**	**-**	**-**	***Lactobacillus***	**-**

### Quality of studies

The quality assessment of the articles was mentioned in a separate table according to the New castle Ottawa Scale (NOS) checklist. The studies that had a score of 7 were considered to have a high quality and were marked in blue color. The quality score describes the strength of the studies in investigating the effect of GDM on the microbiome of women with GDM and their offspring in comparison to the non GDM cases.

The results of the quality assessment of the case-control, cross-sectional, and cohort studies are shown in the **Supplementary Tables 8–10** respectively. The studies that were assessed as high-quality studies are highlighted. All the 22 cohort studies had a good quality according to our assessment (17–20, 23–25, 27, 29–32, 35, 40, 44, 47, 48, 53–55, 59, 63). One cohort study did not meet the NOS criteria and was labeled as unsatisfactory (16). 8 case-control studies had a high quality with a score equal to 7 (22, 26, 43, 45, 52, 57, 64, 65). These studies had matched or adjusted controls regarding some possible confounding factors such as age and BMI. However, other studies that did not adjust the confounding factors acquired lower scores (33, 37, 41, 44, 49, 51). Only 2 cross sectional studies were assessed as high quality with a score equal to 7 (39, 42). The remaining cross sectional studies had a score lower than 7 (21, 36, 38, 46, 50, 56, 60, 61). Three of these studies were pilot studies (28, 60, 62).

## Discussion

### Microbiome of women with GDM

The microbiome is one of the systems that undergo some alterations during pregnancy. Although it is still not very clear whether these changes are a normal consequence of the physiological reactions that take place during pregnancy, or are an independent manifestation that has its own role in contributing to the physiological state, it appears that the microbial communities express a unique pattern during the gestational period. In our study, we are mainly focusing on the microbiome alterations in GDM, where it was found to express a specific signature in GDM cases when compared to the normal groups.

Generally, the microbiome alteration is related to hormonal, immunological, and metabolic changes (66). These changes are expressed as a shift in the microbial composition, manifested by a change in α and β diversity, which measure the diversity of microbial composition in the same sample and among different communities, respectively (66).

Many studies have demonstrated a shift in the gut microbiome during pregnancies towards the energy-producing communities that help in fetus growth, such as the phylum Firmicutes, or the phyla Proteobacteria and Actinobacteria that exert a proinflammatory action by providing protection against infections (67).

According to our systematic review, significant changes in the phyla Firmicutes, Bacteroidetes, Actinobacteria, Proteobacteria, Verrucomicrobia, Fusobacteria and others was observed in the gut microbiome of the women with GDM. The major changes were attributed to the phyla Firmicutes and Bacteroidetes, where they were enriched in most of the studies, although in some studies a decrease in their abundance was seen. The contribution of Firmicutes (butyrate-producing bacteria) and Bacteroidetes (acetate and propionate-producing bacteria) in SCFAs formation and subsequently in adipogenesis supports the idea that their alteration might have a role in obesity and insulin resistance in women with GDM (1). Obesity and glucose intolerance are usually associated with an increase in Firmicutes expression (68, 69).

A decrease in *Bifidobacterium* spp., as beneficial acetate and lactate-producing bacteria, was also seen in women with GDM. This change might be interpreted by the known role of *Bifidobacteria* in the fermentative metabolism, carbohydrates degradation and intracellular uptake of short oligosaccharides (70). Moreover, a significant increase of the genus *Blautia* was a common finding among different studies, which is interesting due to the already proved association between this genus and the glucose intolerance, metabolic disorders, and BMI (69).

*Prevotella* is another genus that was found to be generally increased in the gut of women with GDM. This genus was proposed to have a role in altering the gut permeability by inducing the breakage of mucin oligosaccharides and causing insulin resistance (36, 71).

The role of the gut microbiome changes can be explained either by enhancing the energy production from the diet or by remodeling the metabolic and inflammatory interactive pathways between the host and the microbiome. This is displayed by its association with weight gain, adiposity, insulin resistance, hyperglycemia and a low grade inflammation towards the late phase of pregnancy during third trimester, which is achieved through an increase in glucose and fatty acids absorption as well as by boosting catabolic pathways (17).

Just like the gut microbiome expresses a specific pattern according to different metabolic states, the oral microbiome undergoes similar changes as well. It is known that the oral cavity is the residence site of a wide spectrum of microbial communities due to the exposition of this tract to many ecologic factors, including age, diet, hygiene, oral diseases such as periodontitis, gingivitis and dental caries, hematological spread, connection with the external environment and to a lesser extent to sexual activity (72–74). The oral microflora is mainly composed of *Streptococcus*, and the presentation of some other bacteria differs according to the site of the oral cavity, where *Actinomyces* reside mostly in the supragingival plaque, while the subgingival plaques is mostly predominated by *Prevotella* (74).

These microbial communities share a symbiotic relationship with the host in a normal standard physiological state, where the host provides them with a suitable environment to flourish, and they instead ensure a healthy condition of the oral cavity (73). Pathological conditions might disturb the normal microbial composition.

General changes are usually seen during pregnancy as well, where a shift into a more diverse and enriched microbiome occurs normally especially towards the third trimester of pregnancy (11), where *A. actinomycetemcomitans*, *S. mutans*, *P. gingivalis* and *P. intermedia* were seen to be increased in the saliva microbiome. Regarding the alterations of the oral microbiome of women with GDM, the results were not as much concise in comparison to the gut mircobiome changes according to our study. A scattered pattern of microbial communities’ changes was seen, where a decrease in oral *Streptococci, Lactobacilli*, *and Bifidobacterium* and an increase in *Actinomycetes, Ruminococcaceae* and *Enterobacteriaceae* among other genera was observed. The decrease of *lactobacilli* might leave some negative effects on the oral cavity health, since this bacteria is known by the production of the beneficial organic acid lactates (60). Although not very specific, this pattern of alteration demonstrates a correlation between GDM and the composition of the oral microbiota even though the mechanism by which these bacteria are related to GDM is not yet fully understood.

Similarly, the vaginal microbiome is an assembly of beneficial microorganisms that play their own role in maintaining a healthy status of the vaginal tract, especially due to the importance of this tract in the reproductive functions and its relation with the fetal delivery later. In comparison to the other microbiomes, the vaginal microbiome expresses a less complex composition, with the species *Lactobacillus* predominating in the microbial community with about a 70% frequency (72). Just like the other beneficial microbial communities, *Lactobacillus* is believed to exert a protective function by eliminating the undesired effects of pathologic microbial agents through the acidic environment created by lactic acid production (67).

Being affected by different factors including the hormonal changes during reproductive cycles of women, hygiene, and sexual activity, the vaginal microbiome exhibits a specific pattern during pregnancy, where an enrichment of *Lactobacillus, Clostridiales, Bacterodies*, and *Actinomycetales* takes place. In this study we assessed the vaginal microbiome alterations in women with GDM as well. *Lactobacillus listeri, Lactobacillus amylovorus, and Lactobacillus fructivorans* were significantly associated with GDM. Other genera were also enriched in the vaginal tracts of women with GDM including *Bacteroides, Veillonella, Enterococcus, Enterobacter*, *Fusobacterium*, and *Prevotella* among others.

Since vaginal dysbiosis is known to make the affected women vulnerable for pathological conditions that might lead to adverse pregnancy outcomes such as preterm birth and premature preterm rupture of membranes (PPROM) (67), it is perhaps possible that these changes that occur concurrently with GDM presence might have an effect on the health of women with GDM and their offspring, especially those who are delivered by normal vaginal delivery, which needs to be investigated in future studies.

### Microbiome of offspring to women with GDM

Although it was once believed that the fetal microbial communities are absent before birth and that it is formed at the time of amniotic sac rupture, it was later demonstrated that the fetal microbiome is highly related to that of the mother’s, and that many species of the mother’s microbiome was seen in their offspring tracts including placenta, meconium and fetal membranes (75). Whether the microbiome is transmissible between the mother and the infant is important to better understand the alterations that might occur in the offspring microbiome in pathological states such as GDM. Many hypotheses were proposed to explain the method by which the mother’s microbiome is transferred to their infants, and it was suggested that this process occurs through the placenta and lactation by breastfeeding in later stages. Indeed, the diet of the mother during pregnancy would affect their offspring’s microbiome as well since it will be transmitted to them by placenta (76). The cesarean section mode of delivery was found to lead to a reduced *Bifidobacteria* and *Bacteroides* and an increased *C. difficile* expression, in comparison to the vaginally delivered babies, who were seen to acquire the vaginal microbiome traits of their mothers (77, 78). However, many controversies still surround the effect of delivery mode on the microbiome. It is thought that this effect is only minor and the environmental factors at birth time in addition to the quick conversion to breastfeeding lactation override the initial microbial signature (31). The microbiome takes a role in the future pathophysiological state of the infant (5, 27, 79), and the fact that the initial microbiome species might accompany the infant for quite a prolonged period in the first year shows us the important effect exerted by the microbiome on the gut health not only at the time of birth but in later stages as well (33).

In our review, we evaluated the changes that might occur in the different tracts of the offspring born to women with GDM, and we proved that the offspring microbiome of women with GDM is actually significantly altered. For instance, higher alpha diversity was witnessed in a group of offspring to women with GDM especially in the Bacteroidetes phylum. But in some other groups, a reduced richness in the gut microbiota was seen, which correlated with an increased insulin resistance and proinflammatory markers (42). Some genera that were increased in the cases of babies born to women with GDM include *Rothia* and *Clostridium sensustricto*, which have a role in causing infections and metabolic diseases of childhood (42). However, the genus *Rothia* was also found to be decreased in another study (25), therefore we cannot conclude certainly the correlation between this genus and the presence of GDM.

On a special note, the microflora of the meconium of the offspring was found to undergo very similar changes to the gut microbiome of their mothers who have GDM, including the increase in beta diversity and the enrichment of some phyla such as Proteobacteria towards the last trimester of pregnancy, which encourages us to think of the fact that the microbiome is transmissible between the mothers and their infants (33). Furthermore, microbial species that are related to the development of type 1 diabetes were seen in the gut of the offspring of women with GDM, particularly the *Bacteroides* (32).

*Lactobacillus* that correlates with the early immunological development was also decreased in the gut of offspring to women with GDM, and an increase in *Phascolarctobacterium*, a species that also seen in the gut of mothers with GDM, was witnessed as well (32), in addition to an enrichment in *Lachnospiraceae*, another family that is usually seen in individuals with glycemic dysregulation such as GDM and type 2 diabetes (32). Other changes were observed in the offspring gut as well, and specific strains were seen to correlate with the presence of GDM, such as a decrease in *Prevotella*, a taxonomic biomarker of normal gestational glucose control associated with higher insulin sensitivity (25).

Overall, we found that the changes of the offspring gut microbiome relatively mirror those of their mothers with GDM, which clearly points out an association between this metabolic disorder and its vertical transmission particularly through the microbiome.

Regarding the oral microbiome, it is thought that the microbiome might be transmitted through the maternal gut and the placenta to the oral cavity (37).

Many of the taxa of the neonates microbiome are absent in the mother’s urogenital tract but present in their oral cavity such as *Fusobacterium nucleatum*, that might be transferred *via* hematogenous pathway due to its capacity to adhere to vascular epithelium. It is believed that the oral cavity diseases that might affect the oral microbiome and might be therefore transmitted to the offspring could lead to a preterm birth (80).

It should be noted that infants who were breastfed by women with GDM displayed a different microflora pattern in comparison to those who were breastfed by healthy women, with a notable increase in *Escherichia* and *Parabacteroides*, some pro-inflammatory bacteria that usually reside the tracts of patients with GDM and T2DM (31). According to our review, the phylum Bacteroidetes and the genus *Prevotella* were increased in both the oral microbiome of the offspring and the gut microbiome of the women with GDM, and *Prevotella* was increased in the vaginal microbiome as well, which might indicate a possible association between these manifestations.

The exact mechanism by which the oral microbiome of these infants might interfere with their metabolic functions and predict their future health status is not yet fully understood, but its correlation with the mother’s microbiome indicates a possible chance for the infants to develop metabolic disorders similar to their mothers.

On the other hand, the placenta, being the most important connection way between the pregnant women and their fetuses, was evaluated by some studies and was found to contain some nonpathogenic bacteria in healthy cases including Tenericutes, Fusobacteria, Bacteroidetes, and Firmicutes (4). In the placental microbiome of GDM cases, and although the results were heterogenous among the included conducted studies, changes in the Proteobacteria, Bacteroidetes, Actinobacteria and Firmicutes phyla was observed. For instance, a lower abundance of *Acinetobacter* was observed, which correlates with a metabolic and inflammatory state (39). Meanwhile in another study, the placental microbiome in cases of GDM expressed an increased diversity, and an increased expression of *Ruminococcus* and *Coprococcus* that are involved in the cellobiose and glucose phosphorylation, and in polysaccharides fermentation and SCFAs production, respectively (61). How the placental microbiome is affected by the GDM status of the mother is not very clear, but it is believed that the placenta might interact with the hyperglycemic state induced by GDM through inflammation and oxidative stress, or through the oral-placental route through hematogenous transmission, where many studies proved a similarity between the two microbiome patterns (61). Also, there is a possibility that these species are transmitted to the placenta through vagino-rectal tract to the uterine cavity, and through the lymphatic system of the mother that transfers the microbial species from the gut into blood stream to the placenta (47, 81).

It should be noted that GDM not only correlates with the future metabolic status of the infants, but it also can leave some specific traces in the women’s microbiome as well, where longitudinal studies demonstrated that the microbiome remains altered till even months after delivery, as we showed in our study.

Assessing all these changes is of special importance due to the fact that the infants of mothers with GDM are predisposed to a two- to eightfold increased risk to develop metabolic disorders including impaired insulin sensitivity and type 2 diabetes in addition to obesity later in their life (82), and preventing this from happening is an important concern for clinicians.

### Strengths and limitations

Although the altered signature pattern was significantly observed in our comprehensive systematic review, some limitations prevent us from reaching to a clear defined relationship between GDM and the pattern of microbiome changes. This is in part due to the notable heterogeneity among the studies on different levels. For example, the included ethnicities in most of the studies were from China and Finland, and to a lesser extent from some other countries, which does not allow us to generalize the outcomes, especially that the microbiome is known to be affected by the different genetic, lifestyle and environmental factors that varies among different regions of the world. Another reason is the difference in the criteria used to diagnose GDM, although the diagnosis was mostly given according to IADPSG criteria. The different criteria used to diagnose GDM affected the comparability of the results. Even though the studies mostly included women who had a determined BMI, age, metabolic status and excluded women with specific chronic diseases or medication use, the characteristics of the participants were sometimes different among the studies, which might have affected the final results. This especially applies to the use of antibiotics that leave compositional changes on the microbiome, where the recovery period that is needed after antibiotics treatment is at least 1.5 months, which was variable among the studies included in this review (83). The timing at which the microbiome was analyzed was also different among the articles, especially when the postpartum microbiome was assessed. Regarding the used specimen, the source of the specimen was sometimes different especially in the assessment of the oral microbiome. The number of the included participants was also heterogeneous, where the larger sample size belonged to the studies that assessed the gut microbiome, while lesser studies evaluated the microbial communities in the other tracts. Furthermore, the method of microbial analysis and extraction, amplicon primers and sequencing methods were different among the studies, which led to heterogeneous results.

Regardless of these limitations, all the findings tell us that the correlation between GDM and the microbiome in both women and their offspring is an existing fact. This fact was also addressed in previous studies that proved a GDM-associated microbiota (84, 85), and an increase in *Collinsella* and *Blautia* genera was observed in GDM cases according to Rold et al. (85). However, consistency was not observed among the results and no GDM-specific microbiome patterns could be determined (85). The development of modulating approaches that target the microbiome might have an effect on the prophylaxis and preventing the possible consequences of GDM. Such methods are already being implemented such as using probiotics and prebiotics in women with GDM and assessing their outcome on the metabolic status, and so far the results seem to be promising (1, 86), although we still need more evidence and ongoing studies should further address this outcome in their future researches.

## Conclusion

Gestational Diabetes Mellitus is a common disorder that is getting a special attention recently due to the unwanted outcomes that it leaves on the health of both the women and their offspring. New approaches are being applied in order to develop strategies that prevent GDM and its consequences on the metabolic and physiological state. One of these approaches is through assessment of the alterations of the microbiome in women who had GDM during their pregnancy as well as their offspring. In our systematic review, we concluded a clear existing correlation between GDM and the microbial communities, where specific patterns of alterations in the microbiome was observed in the gut, oral and vaginal tracts of the pregnant women, and in the gut, oral and placental tracts of the offspring as well. These findings, although having some limitations, are promising and encouraging to develop strategies that target the human microbiome in order to develop novel therapeutic plans to treat or prevent GDM using next generation probiotics and parabiotics. Future studies should assess the outcomes and the efficacy of such therapeutic methods.

## Data availability statement

The original contributions presented in the study are included in the article/**Supplementary Material**. Further inquiries can be directed to the corresponding authors.

## Author contributions

SF, H-SE and NS have made substantial contributions to conception and design, acquisition of data, and analysis and interpretation of data. MH was involved in drafting the manuscript and revising it critically for important intellectual content. BL gave the final approval of the version to be published. All authors contributed to the article and approved the submitted version.

## References

[1] Hasain Z Mokhtar NM Kamaruddin NA Ismail NAM Razalli NH Gnanou JV . Gut microbiota and gestational diabetes mellitus: A review of host-gut microbiota interactions and their therapeutic potential. Front Cell Infect Microbiol (2020) 10:188. doi: 10.3389/fcimb.2020.00188 32500037 PMC7243459

[2] Ponzo V Fedele D Goitre I Leone F Lezo A Monzeglio C . Diet-gut microbiota interactions and gestational diabetes mellitus (GDM). Nutrients (2019) 11(2):330. doi: 10.3390/nu11020330 30717458 PMC6413040

[3] Zinina T Tiselko AV Yarmolinskaya MI . The role of intestinal microbiota in the development of complications in pregnant women with gestational diabetes. J Obstet Women's Dis (2020) 69(4):41–50. doi: 10.17816/JOWD69441-50

[4] Yao Y Cai X Chen C Fang H Zhao Y Fei W . The role of microbiomes in pregnant women and offspring: Research progress of recent years. Front Pharmacol (2020) 11:643. doi: 10.3389/fphar.2020.00643 32457628 PMC7225329

[5] Saad MJ Santos A Prada PO . Linking gut microbiota and inflammation to obesity and insulin resistance. Physiol (Bethesda) (2016) 31(4):283–93. doi: 10.1152/physiol.00041.2015 27252163

[6] Taddei CR Cortez RV Mattar R Torloni MR Daher S . Microbiome in normal and pathological pregnancies: A literature overview. Am J Reprod Immunol (2018) 80(2):e12993. doi: 10.1111/aji.12993 29873429

[7] Dabke K Hendrick G Devkota S . The gut microbiome and metabolic syndrome. J Clin Invest (2019) 129(10):4050–7. doi: 10.1172/JCI129194 PMC6763239 31573550

[8] Chen X Devaraj S . Gut microbiome in obesity, metabolic syndrome, and diabetes. Curr Diabetes Rep (2018) 18(12):129. doi: 10.1007/s11892-018-1104-3 30338410

[9] Qin J Li Y Cai Z Li S Zhu J Zhang F . A metagenome-wide association study of gut microbiota in type 2 diabetes. Nature (2012) 490(7418):55–60. doi: 10.1038/nature11450 23023125

[10] Moumne O Hampe ME Montoya-Williams D Carson TL Neu J Francois M . Implications of the vaginal microbiome and potential restorative strategies on maternal health: a narrative review. J Perinat Med (2021) 49(4):402–11. doi: 10.1515/jpm-2020-0367 33554571

[11] Jang H Patoine A Wu TT Castillo DA Xiao J . Oral microflora and pregnancy: a systematic review and meta-analysis. Sci Rep (2021) 11(1):16870. doi: 10.1038/s41598-021-96495-1 34413437 PMC8377136

[12] Pelzer E Gomez-Arango LF Barrett HL Nitert MD . Review: Maternal health and the placental microbiome. Placenta (2017) 54:30–7. doi: 10.1016/j.placenta.2016.12.003 28034467

[13] Dunlop AL Mulle JG Ferranti EP Edwards S Dunn AB Corwin EJ . Maternal microbiome and pregnancy outcomes that impact infant health. Adv Neonat Care (2015) 15(6):377–85. doi: 10.1097/ANC.0000000000000218 PMC4658310 26317856

[14] Nardi GM Grassi R Ndokaj A Antonioni M Jedlinski M Rumi G . Maternal and neonatal oral microbiome developmental patterns and correlated factors: A systematic review-does the apple fall close to the tree? Int J Environ Res Public Health (2021) 18(11):5569. doi: 10.3390/ijerph18115569 34071058 PMC8197112

[15] Wells GA Shea B O’Connell D Peterson J Welch V Losos M . The Newcastle-Ottawa scale (NOS) for assessing the quality of nonrandomised studies in meta-analyses. Oxford (2000).

[16] Solt I Cohavy O . The great obstetrical syndromes and the human microbiome-a new frontier. Rambam Maimonides Med J (2012) 3(2):e0009. doi: 10.5041/RMMJ.10076 23908833 PMC3678810

[17] Koren O Goodrich JK Cullender TC Spor A Laitinen K Backhed HK . Host remodeling of the gut microbiome and metabolic changes during pregnancy. Cell (2012) 150(3):470–80. doi: 10.1016/j.cell.2012.07.008 PMC3505857 22863002

[18] Wang J Zheng J Shi W Du N Xu X Zhang Y . Dysbiosis of maternal and neonatal microbiota associated with gestational diabetes mellitus. Gut (2018) 67(9):1614–25. doi: 10.1136/gutjnl-2018-315988 PMC6109274 29760169

[19] Crusell MKW Hansen T Nielsen T Allin K Ruehlemann M Damm P . Gestational diabetes is associated with an aberrant gut microbiota during pregnancy and postpartum. J Reprod Immunol (2018) 128:56–6. doi: 10.1016/j.jri.2018.05.040

[20] Crusell MKW Brink LR Nielsen T Allin KH Hansen T Damm P . Gestational diabetes and the human salivary microbiota: a longitudinal study during pregnancy and postpartum. BMC Pregnancy Childbirth (2020) 20(1):69. doi: 10.1186/s12884-020-2764-y 32005194 PMC6995204

[21] Cortez RV Taddei CR Sparvoli LG Angelo AGS Padilha M Mattar R . Microbiome and its relation to gestational diabetes. Endocrine (2019) 64(2):254–64. doi: 10.1007/s12020-018-1813-z 30421135

[22] Xu Y Zhang M Zhang J Sun Z Ran L Ban Y . Differential intestinal and oral microbiota features associated with gestational diabetes and maternal inflammation. Am J Physiol Endocrinol Metab (2020) 319(2):E247–e253. doi: 10.1152/ajpendo.00266.2019 31891538

[23] Zhang X Wang P Ma L Guo R Zhang Y Wang P . Differences in the oral and intestinal microbiotas in pregnant women varying in periodontitis and gestational diabetes mellitus conditions. J Oral Microbiol (2021) 13(1):1883382. doi: 10.1080/20002297.2021.1883382 34925709 PMC8676621

[24] Sililas P Huang L Thonusin C Luewan S Chattipakorn N Chattipakorn S . Association between gut microbiota and development of gestational diabetes mellitus. Microorganisms (2021) 9(8):1686. doi: 10.3390/microorganisms9081686 34442765 PMC8400162

[25] Crusell MKW Hansen TH Nielsen T Allin KH Ruhlemann MC Damm P . Comparative studies of the gut microbiota in the offspring of mothers with and without gestational diabetes. Front Cell Infect Microbiol (2020) 10. doi: 10.3389/fcimb.2020.536282 PMC7645212 33194786

[26] Mokkala K Paulin N Houttu N Koivuniemi E Pellonpera O Khan S . Metagenomics analysis of gut microbiota in response to diet intervention and gestational diabetes in overweight and obese women: a randomised, double-blind, placebo-controlled clinical trial. Gut (2021) 70(2):309–18. doi: 10.1136/gutjnl-2020-321643 32839200

[27] Mokkala K Houttu N Vahlberg T Munukka E Ronnemaa T Laitinen K . Gut microbiota aberrations precede diagnosis of gestational diabetes mellitus. Acta Diabetol (2017) 54(12):1147–9. doi: 10.1007/s00592-017-1056-0 28980079

[28] Festa C Drago L Martorelli M Di Marino VP Bitterman O Corleto CC . Flash on gut microbiome in gestational diabetes: a pilot study. New Microbiol (2020) 43(4):195–7. 33135080

[29] Ferrocino I Ponzo V Gambino R Zarovska A Leone F Monzeglio C . Changes in the gut microbiota composition during pregnancy in patients with gestational diabetes mellitus (GDM). Sci Rep (2018) 8:12216. doi: 10.1038/s41598-018-30735-9 30111822 PMC6093919

[30] Di Paola M Seravalli V Paccosi S Linari C Parenti A De Filippo C . Identification of vaginal microbial communities associated with extreme cervical shortening in pregnant women. J Clin Med (2020) 9(11):3621. doi: 10.3390/jcm9113621 33182750 PMC7698214

[31] Ponzo V Ferrocino I Zarovska A Amenta MB Leone F Monzeglio C . The microbiota composition of the offspring of patients with gestational diabetes mellitus (GDM). PloS One (2019) 14(12):e0226545. doi: 10.1371/journal.pone.0226545 31841548 PMC6913919

[32] Soderborg TK Carpenter CM Janssen RC Weir TL Robertson CE Ir D . Gestational diabetes is uniquely associated with altered early seeding of the infant gut microbiota. Front Endocrinol (Lausanne) (2020) 11:603021. 33329403 10.3389/fendo.2020.603021 PMC7729132

[33] Hu JZ Nomura Y Bashir A Fernandez-Hernandez H Itzkowitz S Pei ZH . Diversified microbiota of meconium is affected by maternal diabetes status. PloS One (2013) 8(11). doi: 10.1371/journal.pone.0078257 PMC3819383 24223144

[34] Olomu I Long R Vyas A Luellwitz R Singh P Hoang V . The influence of gestational diabetes on placental microbiome. Placenta (2017) 57:308–8. doi: 10.1016/j.placenta.2017.07.265

[35] Mullins TP Tomsett KI Gallo LA Callaway LK McIntyre HD Dekker Nitert M . Maternal gut microbiota displays minor changes in overweight and obese women with GDM. Nutr Metab Cardiovasc Dis (2021) 31(7):2131–9. doi: 10.1016/j.numecd.2021.03.029 34116892

[36] Fugmann M Breier M Rottenkolber M Banning F Ferrari U Sacco V . The stool microbiota of insulin resistant women with recent gestational diabetes, a high risk group for type 2 diabetes. Sci Rep (2015) 5:13212. doi: 10.1038/srep13212 26279179 PMC4538691

[37] Singh P Rajora P Parihar AS Kaur P Gandhi P Gandhi V . Evaluation of effect of gestational diabetes mellitus on composition of the initial oral microbiota of neonates. Adv Biomed Res (2020) 9(1):78. doi: 10.4103/abr.abr_179_20 33912494 PMC8059455

[38] Hasain Z Raja Ali RA Abdul Razak S Azizan KA El-Omar E Razalli NH . Gut microbiota signature among Asian post-gestational diabetes women linked to macronutrient intakes and metabolic phenotypes. Front Microbiol (2021) 12. doi: 10.3389/fmicb.2021.680622 PMC8270638 34248897

[39] Bassols J Serino M Carreras-Badosa G Burcelin R Blasco-Baque V Lopez-Bermejo A . Gestational diabetes is associated with changes in placental microbiota and microbiome. Pediatr Res (2016) 80(6):777–84. doi: 10.1038/pr.2016.155 27490741

[40] Kuang YS Lu JH Li SH Li JH Yuan MY He JR . Connections between human gut microbiome and gestational diabetes mellitus. Gigascience (2017) 6(8):1–12. doi: 10.1093/gigascience/gix058 PMC5597849 28873967

[41] Yu H Liu Z Dong S . Changes in intestinal flora, TNF-α, l-17, and IL-6 levels in patients with gestational diabetes mellitus. Eur J Inflamm (2018) 16:205873921879355. doi: 10.1177/2058739218793550

[42] Chen T Qin Y Chen M Zhang Y Wang X Dong T . Gestational diabetes mellitus is associated with the neonatal gut microbiota and metabolome. BMC Med (2021) 19(1):120. doi: 10.1186/s12916-021-01991-w 34039350 PMC8157751

[43] Zheng W Xu Q Huang W Yan Q Chen Y Zhang L . Gestational diabetes mellitus is associated with reduced dynamics of gut microbiota during the first half of pregnancy. mSystems (2020) 5(2). doi: 10.1128/mSystems.00109-20 PMC7093821 32209715

[44] Wu Y Bible PW Long S Ming WK Ding W Long Y . Metagenomic analysis reveals gestational diabetes mellitus-related microbial regulators of glucose tolerance. Acta Diabetol (2020) 57(5):569–81. doi: 10.1007/s00592-019-01434-2 31820107

[45] Yao H Xu D Zhu Z Wang G . Gestational diabetes mellitus increases the detection rate and the number of oral bacteria in pregnant women. Med (Baltimore) (2019) 98(11):e14903. doi: 10.1097/MD.0000000000014903 PMC6426525 30882709

[46] Cui MJ Qi C Yang LP Zhang MY Wang HY She GT . A pregnancy complication-dependent change in SIgA-targeted microbiota during third trimester. Food Funct (2020) 11(2):1513–24. doi: 10.1039/C9FO02919B 31994568

[47] Olomu IN Pena-Cortes LC Long RA Long RA Vyas A Krichevskiy O . Elimination of "kitome" and "splashome" contamination results in lack of detection of a unique placental microbiome. BMC Microbiol (2020) 20(1):157. doi: 10.1186/s12866-020-01839-y 32527226 PMC7291729

[48] Wei J Qing Y Zhou H Liu J Qi C Gao J . 16S rRNA gene amplicon sequencing of gut microbiota in gestational diabetes mellitus and their correlation with disease risk factors. J Endocrinol Invest (2021) 45:279–89. doi: 10.1007/s40618-021-01595-4 PMC8308075 34302684

[49] Li XQ Zheng JY Ma XL Zhang B Zhang JY Wang WH . The oral microbiome of pregnant women facilitates gestational diabetes discrimination. J Genet Genomics (2021) 48(1):32–9. doi: 10.1016/j.jgg.2020.11.006 33663937

[50] Li G Yin P Chu S Gao W Cui S Guo S . Correlation analysis between GDM and gut microbial composition in late pregnancy. J Diabetes Res (2021) 2021:8892849. doi: 10.1155/2021/8892849 33628840 PMC7889370

[51] Hou M Li F . Changes of intestinal flora, cellular immune function and inflammatory factors in Chinese advanced maternal age with gestational diabetes mellitus. Acta Med Mediterr (2020) 36(2):1137–42. doi: 10.19193/0393-6384_2020_2_178

[52] Chen T Zhang YQ Zhang YY Shan CJ Zhang YY Fang K . Relationships between gut microbiota, plasma glucose and gestational diabetes mellitus. J Diabetes Invest (2021) 12(4):641–50. doi: 10.1111/jdi.13373 PMC8015828 32702151

[53] Su Y Wang HK Gan XP Chen L Cao YN Cheng DC . Alterations of gut microbiota in gestational diabetes patients during the second trimester of pregnancy in the shanghai han population. J Transl Med (2021) 19(1):366. doi: 10.1186/s12967-021-03040-9 34446048 PMC8394568

[54] Liu H Pan LL Lv S Yang Q Zhang H Chen W . Alterations of gut microbiota and blood lipidome in gestational diabetes mellitus with hyperlipidemia. Front Physiol (2019) 10:1015. doi: 10.3389/fphys.2019.01015 31447702 PMC6691352

[55] Chen F Gan Y Li Y He W Wu W Wang K . Association of gestational diabetes mellitus with changes in gut microbiota composition at the species level. BMC Microbiol (2021) 21(1):147. doi: 10.1186/s12866-021-02207-0 33990174 PMC8122539

[56] Ye G Zhang L Wang M Chen Y Gu S Wang K . The gut microbiota in women suffering from gestational diabetes mellitus with the failure of glycemic control by lifestyle modification. J Diabetes Res (2019) 2019:12. doi: 10.1155/2019/6081248 PMC6854930 31772944

[57] Hu P Chen X Chu X Fan M Ye Y Wang Y . Association of gut microbiota during early pregnancy with risk of incident gestational diabetes mellitus. J Clin Endocrinol Metab (2021) 106(10):e4128–41. doi: 10.1210/clinem/dgab346 34015117

[58] Zhang XH Liao Q Wang F Li D . Association of gestational diabetes mellitus and abnormal vaginal flora with adverse pregnancy outcomes. Medicine (2018) 97(34):e11891. doi: 10.1097/MD.0000000000011891 30142788 PMC6112872

[59] Su M Nie Y Shao R Duan S Jiang Y Wang M . Diversified gut microbiota in newborns of mothers with gestational diabetes mellitus. PloS One (2018) 13(10):e0205695. doi: 10.1371/journal.pone.0205695 30332459 PMC6192631

[60] He ZJ Wu J Xiao B Xiao S Li H Wu K . The initial oral microbiota of neonates among subjects with gestational diabetes mellitus. Front Pediatr (2019) 7: 513. doi: 10.3389/fped.2019.00513 31921726 PMC6914726

[61] Tang N Gan Y Li Y He W Wu W Wang K . The association between gestational diabetes and microbiota in placenta and cord blood. Front Endocrinol (2020) 11:550319. doi: 10.3389/fendo.2020.550319 PMC7609904 33193081

[62] Zheng J Xiao X Zhang Q Mao L Yu M Xu J . The placental microbiota is altered among subjects with gestational diabetes mellitus: A pilot study. Front Physiol (2017) 8: 675. doi: 10.3389/fphys.2017.00675 28932201 PMC5592210

[63] Dong LN Han L Duan T Lin S Li J Liu X . Integrated microbiome-metabolome analysis reveals novel associations between fecal microbiota and hyperglycemia-related changes of plasma metabolome in gestational diabetes mellitus. Rsc Adv (2020) 10(4):2027–36. doi: 10.1039/C9RA07799E PMC9048209 35494569

[64] Wang X Liu H Li Y Huang S Zhang L Cao C . Altered gut bacterial and metabolic signatures and their interaction in gestational diabetes mellitus. Gut Microbes (2020) 12(1):1–13. doi: 10.1080/19490976.2020.1840765 PMC7714515 33222612

[65] Ma S You Y Huang L Long S Zhang J Guo C . Alterations in gut microbiota of gestational diabetes patients during the first trimester of pregnancy. Front Cell Infect Microbiol (2020) 10:58. doi: 10.3389/fcimb.2020.00058 32175285 PMC7056672

[66] Neuman H Koren O . The pregnancy microbiome. Nestle Nutr Inst Workshop Ser (2017) 88:1–9. doi: 10.1159/000455207 28346919

[67] De Siena M Laterza L Matteo MV Mignini I Schepis T Rizzatti G . Gut and reproductive tract microbiota adaptation during pregnancy: New insights for pregnancy-related complications and therapy. Microorganisms (2021) 9(3):473. doi: 10.3390/microorganisms9030473 33668738 PMC7996258

[68] Le Chatelier E Nielsen T Qin J Prifti E Hildebrand F Falony G . Richness of human gut microbiome correlates with metabolic markers. Nature (2013) 500(7464):541–6. doi: 10.1038/nature12506 23985870

[69] Egshatyan L Kashtanova D Popenko A Tkacheva O Tyakht A Alexeev D . Gut microbiota and diet in patients with different glucose tolerance. Endocr Connect (2016) 5(1):1–9. doi: 10.1530/EC-15-0094 26555712 PMC4674628

[70] Rivière A Selak M Lantin D Leroy F De Vuyst . Bifidobacteria and butyrate-producing colon bacteria: Importance and strategies for their stimulation in the human gut. Front Microbiol (2016) 7:979. doi: 10.3389/fmicb.2016.00979 27446020 PMC4923077

[71] Ottosson F Brunkwall L Ericson U Nilsson PM Almgren P Fernandez C . Connection between BMI-related plasma metabolite profile and gut microbiota. J Clin Endocrinol Metab (2018) 103(4):1491–501. doi: 10.1210/jc.2017-02114 29409054

[72] Cobb CM Kelly PJ Williams KB Babbar S Angolkar M Derman RJ . The oral microbiome and adverse pregnancy outcomes. Int J Womens Health (2017) 9:551–9. doi: 10.2147/IJWH.S142730 PMC5557618 28848365

[73] Zarco MF Vess TJ Ginsburg GS . The oral microbiome in health and disease and the potential impact on personalized dental medicine. Oral Dis (2012) 18(2):109–20. doi: 10.1111/j.1601-0825.2011.01851.x 21902769

[74] Gao L Xu T Huang G Jiang S Gu Y Chen F . Oral microbiomes: more and more importance in oral cavity and whole body. Protein Cell (2018) 9(5):488–500. doi: 10.1007/s13238-018-0548-1 29736705 PMC5960472

[75] Gohir W Ratcliffe EM Sloboda DM . Of the bugs that shape us: maternal obesity, the gut microbiome, and long-term disease risk. Pediatr Res (2015) 77(1):196–204. doi: 10.1038/pr.2014.169 25314580

[76] Lv Y Yan Z Zhao X Gang X He G Sun L . The effects of gut microbiota on metabolic outcomes in pregnant women and their offspring. Food Funct (2018) 9(9):4537–47. doi: 10.1039/C8FO00601F 30101246

[77] Penders J Thijs C Vink C Stelma FF Snijders B Kummeling I . Factors influencing the composition of the intestinal microbiota in early infancy. Pediatrics (2006) 118(2):511–21. doi: 10.1542/peds.2005-2824 16882802

[78] Dominguez-Bello MG Costello EK Contreras M Magris M Hidalgo G Fierer N . Delivery mode shapes the acquisition and structure of the initial microbiota across multiple body habitats in newborns. Proc Natl Acad Sci U.S.A. (2010) 107(26):11971–5. doi: 10.1073/pnas.1002601107 PMC2900693 20566857

[79] Zhou L Xiao X . The role of gut microbiota in the effects of maternal obesity during pregnancy on offspring metabolism. Biosci Rep (2018) 38(2):BSR20171234. doi: 10.1042/BSR20171234 29208770 PMC5897743

[80] Aagaard K Ma J Antony KM Ganu R Petrosino J Versalovic J . The placenta harbors a unique microbiome. Sci Transl Med (2014) 6(237):237ra65. doi: 10.1126/scitranslmed.3008599 PMC4929217 24848255

[81] Stinson LF Payne MS Keelan JA . Planting the seed: Origins, composition, and postnatal health significance of the fetal gastrointestinal microbiota. Crit Rev Microbiol (2017) 43(3):352–69. doi: 10.1080/1040841X.2016.1211088 27931152

[82] Damm P Houshmand-Oeregaard A Kelstrup L Lauenborg J Mathiesen ER Clausen TD . Gestational diabetes mellitus and long-term consequences for mother and offspring: a view from Denmark. Diabetologia (2016) 59(7):1396–9. doi: 10.1007/s00125-016-3985-5 27174368

[83] Palleja A Mikkelsen KH Forslund SK Kashani A Allin KH Nielsen T . Recovery of gut microbiota of healthy adults following antibiotic exposure. Nat Microbiol (2018) 3(11):1255–65. doi: 10.1038/s41564-018-0257-9 30349083

[84] Medici Dualib P Ogassavara J Mattar R Mariko Koga da Silva E Atala Dib S de Almeida Pititto B . Gut microbiota and gestational diabetes mellitus: A systematic review. Diabetes Res Clin Pract (2021) 180:109078. doi: 10.1016/j.diabres.2021.109078 34599971

[85] Rold LS Bundgaard-Nielsen C Niemann Holm-Jacobsen J Glud Ovesen P Leutscher P Hagstrøm S . Characteristics of the gut microbiome in women with gestational diabetes mellitus: A systematic review. PloS One (2022) 17(1):e0262618. doi: 10.1371/journal.pone.0262618 35025980 PMC8757951

[86] Han MM Sun J-F Su X-H Peng Y-F Goyal H Wu C-H . Probiotics improve glucose and lipid metabolism in pregnant women: a meta-analysis. Ann Transl Med (2019) 7(5):99. doi: 10.21037/atm.2019.01.61 31019949 PMC6462661

