# Supplementary material for: Microbiome alterations in women with gestational diabetes mellitus and their offspring: A systematic review
Source: Front Endocrinol (Lausanne). 2022 Dec 8;13:1060488. doi: 10.3389/fendo.2022.1060488 (PMC9772279; doi:10.3389/fendo.2022.1060488)
Supplement: Supplementary file 1 [file Table_1.docx]

**Supplementary Table 1: The extracted data related to the gut microbiome profile in GDM patients**

| Gut microbiota profile in GDM patients in comparison to controls (phylum/ order/ family/ genus) | Method of microbial analysis | Analyzed microbiome  And  Sample source | Method of diagnosis of GDM | Participants characteristics | Timing of analysis | Total sample size | Country and/or ethnicity | Study type | Study year |  |
| --- | --- | --- | --- | --- | --- | --- | --- | --- | --- | --- |
| Skewed prevalence for multiple genera in either gestational diabetes or the healthy state, suggesting changes in microbial composition for the oral, vaginal, and rectal compartments in women with gestational diabetes when compared with healthy pregnancies | 16S rRNA sequencing | Gut microbiome | - | - | Third-trimester | Total sample n= 29 | Israel | Cohort | Published:  April, 2012 | Solt, I. et al. [16] |
| Shift from T1 to T3 in β-diversity in both groups  low phylogenetic diversity in GDM patients | 16S rRNA sequencing | Gut microbiome  Fecal sample | - | Women who were previously recruited for a prospective, randomized mother-infant nutrition study in Finland | -At T1 (13.84 ± 0.16 weeks)  -at T3 (33.72 ± 0.12 weeks) | Total sample n= 91  -With GDM n=15  -Without GDM n=76 | Finland | Cohort | Published:  August 2, 2012 | Koren, O. et al. [17] |
| - Family:  ↑ *Ruminococcaceae* | 16S rRNA sequencing | Gut microbiome  Fecal sample | IADPSG criteria | -Included: ≤ 17 weeks of gestation, BMI ≥ 25, age 18–45 years.  - Excluded: chronic metabolic and gastrointestinal diseases including celiac disease, infammatory bowel diseases and diabetes | Mean of 12.9 (SD 2.5) weeks of gestation | Total sample n= 75  without GDM at early pregnancy  - After testing for GDM at a mean of 25.5 weeks of gestation:  -Developed GDM n= 15  -Did not develop GDM n= 60 | Finland | Cohort | Published: 4 October 2017 | Mokkala, K. et al. [27] |
| -Genus:  ↑ *Bacteroides*  *↑ Parabacteroides*  -Species:  ↑ *Parabacteroides distasonis*  *↑ Klebsiella variicola*  *↑ Catenibacterium mitsuokai*  *↑ Coprococcus comes*  *↑ Citrobacter*  *↓ Bifidobacterium spp.*  (*B. pseudocatenulatum, B. animalis,* and 1 unclassified MLG)  ↓ *Eubacterium spp. (E. siraeum, E. eligens, 2* unclassified *Eubacterium* MLG*s)*  *↓ Roseburia spp.* | Metagenomic shotgun sequencing | Gut microbiome  Fecal sample | Chinese diagnostic criteria | Without pre-pregnancy diabetes, hypertension, thyroid disorders, asthma, lipid metabolic disorders, inflammatory bowel disease, irritable bowel syndrome, and celiac disease; any antibiotic treatment 1 month or any probiotics 2 weeks before sample collection | Second trimester | Total sample n= 287 were eligible  - With GDM n=43  -Without GDM n=81 (randomly selected) | China | Cohort | between 1 August 2012 and 31 August 2013  Published:  31 July 2017 | Kuanget, Y. S. et al. [40] |
| -No obvious variation in intestinal microbiota was shown.  -Correlated with GDM across multiple sample types similarly between maternal and neonatal samples:  *Blautia*  *Coprococcus*  *Roseburia*  *Sutterella* | 16S rRNA gene and metagenomic sequencing | Gut microbiome    Fecal samples | According to the results of OGTT | Chinese pregnant women and neonates  The vast majority of the neonatal samples were collected from caesarean delivery (76 C-sections vs 17 vaginal deliveries) | 1–2 days before delivery | Total sample n= 486  (n=140 neonates and n=346 pregnant women) with n=1062 total samples collected  n=581 maternal samples(n=175 oral, n=147 intestinal and n=259 vaginal) | Chinese women | Cohort | Published:  14 May 2018 | Wang, J. et al. [18] |
| - From the phylym Actinobacteria:  Genus: Collinsella ↑  Parent family: Coriobacteriaceae ↑  parent order: Coriobacteriales ↑  Genus: Rothia ↑  Parent family: Micrococcaceae ↑  Parent order: Actinomycetales ↑  Genus: Actinomyces ↑  Order: Actinomycetales ↑  - From the phylum Proteobacteria:  Genus: Desulfovibrio ↑  - From the phylum Firmicutes:  Genus Leuconostoc ↑  Parent family: Leuconostocaceae ↑  Genus Granulicatella ↑  Genus Mogibacterium↑  - OTUs:  ↑ Blautia, Ruminococcus, Faecalibacterium, Anaerotruncu  ↓ Acetivibrio , Intestinimonas, Erysipelotrichaceae incertae sedis , Isobaculum, Butyricicoccus, Clostridium IV, Clostridium XVIII, Oscillibacter, Ruminococcus, Bacteroides, Veillonella, Suterella, Faecalibacterium | 16S rRNA sequencing. | Gut microbiome  Fecal sample | IADPSG criteria | - Included: singleton pregnant, Danish white origin , without diagnosed pre-eclampsia at the time of inclusion, multipara women with a previous normoglycaemic pregnancy  - Excluded:  antibiotics within a period of 2 months before the first visit | Third trimester (27–33 gestational weeks) | Total sample n= 209  -With GDM n=50  -Without GDM n= 157  - n=2 with uncertain diabetes status | Denmark  Danish white origin | Cohort | January 2014 to February 2015  Published:  15 May 2018 | Crusell, M.K.W. et al. [19] |
| - Phylum:  ↑ Firmicutes  ↓ Bacteroidetes  ↓ Actinobacteria  - Genus:  ↓ *Bacteroides, Collinsella and Rikenellaceae*  ↑ *Blautia, Butyricicoccus, Clostridium,* *Coprococcus, Dorea, Faecalibacterium, L−Ruminococcus, Lachnospiraceae* | 16S amplicon-based sequencing | Gut microbiome  Fecal sample | International criteria | - Included: overweight women, with excessive fat intake and lower than recommended fber consumption  - Excluded: twin pregnancy, use of prebiotics/probiotics, antibiotics or any drug during pregnancy, any pathological conditions before or during pregnancy,  no compliance to the study protocol | - At enrolment (24–28 weeks)  - At 38 weeks of gestational age | Total sample n= 50 with GDM  N= 9 lost in follow up  N= 7 gave birth before 38 weeks and gave samples at 37^th^ week | Turin, Italy  Caucasian race | Cohort study that includes a dietary intervention aspect | From April 2016  Published:  15 August 2018 | Ferrocino, I. et al. [29] |
| ↓ *Bifidobacterium*  *↓ Lactobacillus*  *↓ Bacteroides* | 16S rRNA sequencing | Gut microbiome  Fecal sample | Guidelines for the Prevention and Treatment of Type 2 Diabetes in China | - Included: women aged 21– 35years first or second time pregnant at 24–29 weeks gestation  -Excluded: severe heart, lung, or hematological diseases, severe intestinal diseases or history of intestinal surgery, use of antibiotics or hormone drugs for long time | at 24–29 weeks gestation | Total sample n= 140  -With GDM n=80  -Without GDM n=60 | China | Case-control | From March 2016 to March 2017  Published: 2018 | Yu H. et al. [41] |
| - Phylum: (no statistical significance)  ↑ Firmicutes  - Genus: (no statistical significance)  ↑ *Ruminococcus*  *↑ Eubacterium*  *↑ Prevotella* | 16S rRNA sequencing | Gut microbiome  Fecal sample | WHO  Criteria | -Included: singleton pregnancy, live fetus  - Excluded: multiple pregnancy, fetal death, autoimmune diseases, pre-existing diabetes, uterine malformations, pregnancy resulting from in vitro fertilization, placental abruption, infections, cancer, or any other systemic disease | Third trimester of gestation (28–36 weeks) | Total sample n= 68  -With GDM n=26  -Without GDM n=42 | São Paulo, Brazil | Cross-sectional study | Between 2014 and 2016  Published:  12 November 2018 | Cortez, R. V. et al. [21] |
| - Phylum:  ↑ Verrucomicrobia  - Genus:  In GDM only cohort:  ↑ *Roseburia, Bacteroides and Lachnospiraceae*  In GDM plus hyperlipidemia cohort:  ↑ *Bacteroides, Roseburia and Prevotella*  - Taxa in GDM plus hyperlipidemia cohort:  ↑ *Streptococcus, Faecalibacterium, Veillonella, Prevotella, Haemophilus and Actinomyces* | 16S rRNA sequencing | Gut microbiome  Fecal sample | IADPSG criteria | - Included: previously diagnosed as healthy, with GDM only or with GDM plus hyperlipidemia  - Excluded: antibiotic use in the previous 3 months or active smoking | Third trimester (27–33 weeks) | Total sample n= 45  -control n=11  -With GDM only n=11  - With only hyperlipidemia n=11  - With GDM plus hyperlipidemia n=12 | Wuxi, China | Cohort | During September 2017 to March 2018  Published:  06 August 2019 | Liu, H. et al. [54] |
| - No significant difference between the N group and the GDM1 group  - In GDM2:  ↓ Faecalibacterium  ↓ Subdoligranulum  ↑ Blautia and Eubacterium_hallii | 16S rRNA sequencing | Gut microbiome  Fecal sample | IADPSG criteria | - Included: singleton pregnant women  - Excluded:  pre-pregnancy diabetes mellitus, family history of diabetes mellitus  hypertension, Cushing syndrome, hyperthyroidism, hyperlipidemia, genetic disease, women with inflammatory bowel disease, irritable bowel syndrome, celiac disease, using antibiotic or probiotics within one month | 24-28 gestational weeks | Total sample n=52  - Without GDM n=16  -With GDM n=36  Divided into:  -successful glycemic control (GDM1 group, n = 24)  - failure of glycemic control (GDM2 group, n = 12) | Zhejiang, China | Cross sectional | From September 1, 2016 to November 28, 2017  Published:  23 October 2019 | Ye, G. et al. [65] |
| ↑ *Bacteroides* genus (species: *Bacteroides dorei and Bacteroide*s sp. 3_1_3FAA) | Whole-metagenome shotgun sequencing  and  Q-PCR (quantitative polymerase chain reaction) | Gut microbiome  Fecal sample | ADA criteria | - Included:  Women who received regular prenatal care, took oral glucose tolerance test (OGTT), and delivered a single baby  -Excluded:  use of prebiotics/ probiotics, antibiotic within 6 months, gastrointestinal disease, established type 1 or type 2 diabetes; established hyperlipidemia, hypertension, or metabolic syndrome before pregnancy; a history of severe systemic disease | Third trimester | In the case-control study:  - With GDM n=23  - Without GDM n=26  From the cohort-based study:  N=150  -with GDM n=37  - without GDM n=113 | China  Chinese Han women | Case-Control  (Some data are based upon another cohort) | between September 1, 2016, and December 31, 2016  Published:  9 December 2019 | Wu, Y. et al. [44] |
| -Significant β-diversity  - Class: ↑ *Gammaproteobacteria*  - Genus: ↑ *Haemophilus* | 16S rRNA sequencing | Gut microbiome  Fecal sample | WHO criteria | Excluded if: Age <18, other pregnancy complications, multiple pregnancy, pre-pregnancy diabetes, stress and anxiety, depression, probiotics and antibiotics in last 2 weeks, GI discomfort, history of smoking | Third trimester | Total sample n=61  -With GDM n=30  -Without GDM n=31 | Zhengzhou, China | case-control | From January to August, 2018  Published:  January 1, 2020 | Xu, Y. et al. [22] |
| -Genus:  ↓ *Blautia*  *↑ Phascolarctobacterium*  *↑ Alistipes*  *↑ Parabacteroides*  *↑ Eubacterium*  *↑ coprostanoligenes group*  *↑ Oscillibacter*  *↑ Paraprevotella*  *↑ Ruminococcaceae* | 16S rRNA sequencing | Gut microbiome  Fecal sample | IADPSG criteria | Women with known pre-existing diabetes, chronic or serious acute infections, abnormal liver or kidney function, cardiovascular hematological diseases were excluded | 24th to 26th weeks of gestation | Total sample n= 40  -With GDM  n= 20  -Without GDM n= 20 | Shanxi Province, China | Cohort | Between May and September of 2018  Published:  9 January 2020 | Dong, L. N. et al. [60] |
| - No significant total faecal microbiota separation among the four groups  - Significant separation in β-diversity  - Phylum: ↑ Bacteroidetes  - Greater SIgA-binding diversity (of *Lactococcus, Escherichia, Streptococcus, Enterococcus, Lactobacillus)* | 16S rRNA sequencing for both total faecal microbiota and SIgA-targeted microbiota | Gut microbiome  Fecal sample | Hospital diagnostic criteria (fasting plasma glucose ≥5.1 mmol L−1, GHbA1c ≥6%) | Did not receive any antibiotic treatment for 1 month and did not take probiotics for 2 weeks before sample collection | Third trimester  (36.35 ± 4.12 weeks) | Total sample n= 91  -With GDM  n = 21    -With gestational hypertension n = 20  -With dyslipidemia n=14  -healthy n = 36 | Changzhou, China | Cross- sectional | Between 15 March 2018 and 15 May 2018  Published:  13 January 2020 | Cui, M. J. et al. [46] |
| -Phylum:  ↓ Firmicutes in both AMA and NMA groups  ↑ Bacteroidetes and Proteobacteria in both AMA and NMA groups  ↑ Fusobacteria, ↑ Verrucomicrobia,  ↑ Actinobacteria in AMA in comparison to other groups | 16S rRNA sequencing | Gut microbiome  Fecal sample | Fasting blood glucose 5.1mmol/l, 1 hour blood glucose 10.0mmol/l, 2 hours blood glucose 8.5mmol/l | Included:  Not diagnosed with diabetes before pregnancy; no antibiotics, probiotics, acute or chronic gastroenteritis, irritable bowel syndrome, gastrointestinal surgery in the past 1 month; no drugs that interfere with glucose metabolism were used during pregnancy | at the 24-28 week of gestation | Total sample n=111  -With GDM n= 61  Divided into:  -AMA group (n=29) with age > 35  -NMA group (n=32) with age < 35  -Without GDM n=50 | Beijing, China | Case-control | From January 2017 to June 2018  Accepted: January 20, 2020 | Hou, M. et al. [51] |
| Genus:  ↑ *Eisenbergiella*  *↑ Tyzzerella 4*  *↑ Lachnospiraceae NK4A136* | 16S rRNA sequencing | Gut microbiome  Fecal sample | IADPSG  criteria | Singleton pregnancy and natural conception, diabetes-free at recruitment, without diseases that might affect microbiome composition or glucose metabolism, had not received any antibiotic treatment throughout the current pregnancy; no acute infection 2 weeks before sample collection | Early pregnancy (10–15 weeks) | Recruited n= 828  With GDM n=112  Matched cases with complete clinical data:  -With GDM n=98  -Without GDM n=98 | South China | Nested case-control study  (based upon an early pregnancy follow-up cohort) | From March 2017 to 2018  Published:  27 February 2020 | Ma, S. J. et al. [63] |
| - Genus:  - in T1:  Difference in: *Prevotella*  *Coprococcus Streptococcus Peptococcus Desulfovibrio Intestinimonas Veillonella*  *Parent taxa of Streptococcus*  - In T2:  ↑ *Holdemania*  *↑ Megasphaera*  *↑ Eggerthella*  - In both T1 and T2:  ↓ *Coprococcus*  *↓ Streptococcus*  *↑ Megasphaera*  *↑ Eggerthella* | 16S rRNA sequencing | Gut microbiome  Fecal sample | ADA  criteria | - Included: Women at 18 to 45 years of age with a singleton pregnancy  - Excluded:  chronic medical conditions, reported use of antibiotics or medications that would affect gastric or intestinal microbiota within the 2 months prior to entry | First trimester (T1) to the second trimester (T2) | Total sample n= 134  -With GDM n= 31  -Without GDM  n= 103 | Beijing, China | Nested case-control study | From July 2017 to December 2018  Published:  24 March 2020 | Zheng, W. et al. [43] |
| - No differences were observed  - Only Firmicutes/ Bacteroidetes ratio was higher - in women with early onset GDM compared with those without in late pregnancy  - In women with mid-pregnancy onset GDM, only one bacterial species (*Roseburia hominis*) changed | - Metagenomic sequencing  (Quantitative real-time PCR) | Gut microbiome  Fecal sample | -National guidelines  - IADPSG | - Included: overweight and obese pregnant women, prepregnancy body mass index (BMI) ≥25, early pregnancy (<18 weeks of gestation)  - Excluded:  GDM diagnosed before the first study visit, multifetal pregnancy, Presence of metabolic/ inflammatory diseases, did not provide faecal samples at both time points of their pregnancy, used antibiotics within 8 weeks before the stool sampling, medication for the treatment of GDM | Early (mean gestational weeks 13.9)  Late  (mean gestational weeks 35.2) | Total sample  n= 270 after exclusion  According to national criteria:  - Without GDM n= 203  -All GDM cases n= 67  -Early GDM onset n= 14  -Mid pregnancy GDM onset n=53  - Confiremed Mid pregnancy onset: n=16  According to IADPSG:  - Without GDM n= 167  -All GDM cases n= 103  -Early GDM onset n= 31  -Mid pregnancy GDM onset n= 72  - Confirmed Mid pregnancy onset: n=11 | Southwest Finland | Case-control part of a  mother–infant dietary single-center intervention trial | Published:  24 august 2020 | Mokkala, K.  et al. [26] |
| ↓ *Rothia*  *↓ Actinomyces*  *↓ Bifidobacterium*  *↓ Adlercreutzia*  *↓ Coriobacteriaceae*  *↓ Lachnospiraceae*  Family:  *↑ Enterobacteriaceae*  *↑ Ruminococcaceae*  *↑ Veillonellaceae* | 16S rRNA sequencing | Gut microbiome  Fecal sample | IADPS criteria | -Excluded: if received infertility treatments such as in-vitro fertilization and intrauterine insemination, reported severe chronic or infectious diseases (e.g., cancer, HIV, or tuberculosis) | At 24-28 weeks of pregnancy | Total sample n=402  - With GDM n=201  -Without GDM n=201 matched controls | Shuangliu District of Chengdu, China | Nested case-control study | From March 2017 to August 2019  Published: 29 July 2020 | Hu, P. et al. [64] |
| - Phylum: no significant reduction in Bacteroidetes and Firmicutes  - Genus and species : ↑ *of Bacteroides caccae, Bacteroides massiliensis,*  *Bacteroides thetaiotaomicron*  ↓ of *Bacteroides vulgatus, Eubacterium eligens, Lactobacillus rogosae, and Prevotella copri* | 16S rRNA sequencing | Gut microbiome  Fecal sample | IADPS criteria | - Included: Caucasian women aged ≥18 years  - Excluded:  antibiotics, probiotics, symbiotics, metformin use during pregnancy,  obesity, twin pregnancy, pre-gesta tional diabetes, inflammatory bowel diseases | Third trimester  At 34-36 gestational weeks | Total sample n= 29  - With GDM n=14  - Without GDM n=15  N= 9 were excluded from all due to low DNA quality/ quantity  After exclusion:  - with GDM n=10  - without GDM n=10 | Italy  Caucasian women | Pilot study | Published:  31 October 2020 | Festa, C.  Et al. [28] |
| ↑(OTUs) of the family *Lachnospiraceae*  ↓OTUs of the families *Enterobacteriaceae and Ruminococcaceae* | 16S rRNA sequencing | Gut microbiome  Fecal sample | IADPSG criteria | - Have no other complications of pregnancy, diarrhea, and other gastrointestinal symptoms , the gestation fetuses have no chromosomal and structural abnormalities, did not take any antibiotics, probiotics, or prebiotics within 1 month prior to sampling | - At 12 weeks’ gestation  - At 24–28th gestational weeks | Total sample n= 107  -With GDM n=59  -Without GDM n=48 | Chongqing, China | Case- Control | Published: November 2020 | Wang, X. et al. [62] |
| Phylum in the periodontitis + GDM group:  ↓ Firmicutes  ↑ Bacteroidetes compared to healthy controls in intestinal  In the GDM group:  ↑ Defluviitaleaceae  ↑ Lachnospiraceae  ↑ Paracaedibacteraceae | 16S rRNA sequencing | Gut microbiome  Fecal sample | IADPSG criteria | Aged from 20 to 45 years.  Without digestive system diseases, metabolic diseases, or immune system diseases and tumors; had not taken antibiotics or probiotics within the preceding 3 months | In the second trimester (20–28 weeks) | Total sample n=69  - periodontitis n = 28  -With GDM n=7  - periodontitis + GDM n = 7  - periodontitis- and without GDM n = 27 | China | Cohort | Published: 09 Feb 2021 | Zhang, X. et al. [23] |
| -Phylum:  ↓ Bacteroides  ↑ Firmicutes  ↑ Firmicutes/Bacteroides ratio | 16S rRNA sequencing | Gut microbiome  Fecal sample | IADPSG criteria | 52 singleton pregnant women without: prepregnancy diabetes, history of diseases of the digestive tract in the past 4 weeks; use of antibiotics or intestinal probiotics in the past 4 weeks; abnormal routine stool examination | Third trimester at >28 weeks of gestation | Total sample n= 52  -With GDM n=23  divided into a longer gestational week (LG group, n = 12) and shorter gestational week (G group, n = 11)  -Without GDM n= 29 | Zhengzhou, China | Cross sectional | From October 1, 2018, to December 30, 2019  Published:  10 February 2021 | Li, G. et al. [50] |
| - Phylum: ↑ Bacteroidetes  ↓ Firmicutes  ↓ Actinobacteria  - Genus:  Of the phylum Bacetroidetes:  ↑ *Bacteroides, Butyricimonas, Odoribacter*  *Rikenellaceae*  - Of the phylum Proteobacteria:  ↑ *Campylobacter Sutterella*  - Of the phylum  Actinobacterias:  ↑ *Atopobium*  - Of phylum Firmicutes:  ↑ *Dialister*  *↑ Enterococcaceae*  *↓ Gemmiger*  *↓ Oscillospira*  *↓* unassigned genera of *Clostridiales*  ↓ *Ruminococcaceae*  *↓ Lachnospiracea* | 16S rRNA sequencing | Gut microbiome  Fecal sample | IADPSG criteria | Excluded:  prepregnancy diabetes or other prediagnosed metabolic diseases,  diagnosed with GDM based on fasting glucose levels at enrollment, treatment with antibiotics within 3 months before sample collection, chronic diseases requiring medication | Second trimester  between 22 and 24 weeks of pregnancy before diagnosis of GDM | Total sample n= 330  -With GDM n=110  -Without GDM n=220 | Nanjing, Jiangsu Province, China  Unrelated ethnic Han Chinese | Case–control study | between 2017 and 2018  Published:  4 April 2021 | Chen, T. Et al. [52] |
| ↓ 42 genera (including Prevotella and Romboutsia)  ↑ *Corynebacterium spp. (Corynebacterium appendicis, Corynebacterium coyleae, Corynebacterium durum, Corynebacterium frankenforstense, Corynebacterium freneyi, Corynebacterium glaucum, Corynebacterium kroppenstedtii, Corynebacterium xerosis)*  ↑ *Lactobacillus spp. (Lactobacillus ceti, Lactobacillus sanfranciscensis, Lactobacillus vaccinostercus)*  ↑ *Blautia hydrogenotrophica*  *↓ Bifidobacterium spp*  *↓ Eubacterium spp.* | 16S rRNA sequencing | Gut microbiome  Fecal sample | IADPSG criteria | - Included: pregnant women of 22–45 years of age  -Excluded: taking probiotics, use of antibiotics or other drugs within 1 month, complications of delivery pregnancy-induced hypertension, intestinal diseases, acute gastroenteritis, autoimmune, thyroid dysfunction, liver and kidney disease | third trimester (24–28 gestation weeks) | Total sample n=58  -With GDM n=30  -Without GDM n=28 | China | Cohort | Published:  14 May 2021 | Chen, F. et al. [61] |
| - At 16 weeks gestation: no significant differences  -At 28 weeks gestation:  Phyla Firmicutes and Bacteroidetes in similar abundances in both groups.  -Genus: *Eggerthella* ↑ | 16S rRNA sequencing | Gut microbiome  Fecal sample | IADPSG criteria | -Included: Overweight and obese women, defined by a pre-pregnancy BMI >25 kg/m2 , age >18 years, singleton pregnancy  -Excluded: pre-existing diabetes mellitus, known probiotic intake and medications that could affect glucose metabolism | At 16 and 28-weeks’ gestation | Total sample n= 58  -With GDM  n= 29  -Without GDM n=29 | Brisbane, Australia | Sub cohort | Based on the SPRING study (the Study of Probiotics IN the prevention of Gestational diabetes) that started enrolling in 2012  Published: June 2021 | Mullins, T. P. et al. [35] |
| ↑ *Clostridiales*  *↑ Clostridia*  *↑ Firmicutes*  -Species:  ↑ *Ruminococcus bromii*  *↑ Clostridium colinum*  *↑ Streptococcus infantis* | 16S rRNA sequencing | Gut microbiome  Fecal sample | IADPSG/ WHO 2013 criteria | -Included: 20–40 years of age, BMI≤28 kg/m2, had not received any antibiotic 1 month before sample collection; had not taken any probiotics 2 weeks before sample collection  -Excluded: diabetes, hypertension, high cholesterol, thyroid disorders, asthma, fatty liver disease, inflammatory gastroenteritis, irritable bowel syndrome, cardiac, liver, kidney diseases, psychiatric disorders, alcohol abuse, smoking, HIV, malignancy, illicit drug use, autoimmune or endocrine diseases | at 24–28 weeks of gestational age | Total sample n= 46 stool samples  (n=19 with GDM and n=27 without GDM)  After excluding n=9 due to insufficient stool sample + n=4 for exclusion criteria:  Total N=33  -With GDM n=15  -Without GDM n=18 | China | Prospective study (cohort) | From February 2018 to May 2019  Published:  24 July 2021 | Wei, J. et al. [48] |
| ↑ Firmicutes/Bacteroidetes (F/B) ratio at before delivery | 16S rRNA sequencing | Gut microbiome  Fecal sample | NDDG criteria | - Included: Singleton pregnant women  -Excluded: underlying medical diseases prior to pregnancy, multifetal gestation, fetal or neonatal anomaly, receiving corticosteroids, beta-blockers, and antibiotics at the time of GDM diagnosis, incomplete data or unknown pregnancy outcomes | - At (24–28 weeks of gestation)  - Before delivery (≥37 weeks of gestation) | Total sample n=88 and their 88 offspring  -With GDM n=49  -Without GDM n= 39 | Chiang Mai, Thailand | Longitudinal prospective | August 2019 to February 2020  Published:  8 August 2021 | Sililas, P. et al. [24] |
| - Phylum:  ↑ Bacteroidetes  ↓ Proteobacteria  ↓ Actinobacteria  ↓ Verrucomicrobia  ↓ Tenericutes  - Genus:  ↑ *Bacteroides*  *↑ Incertae sedis*  *↑ Citrobacter*  ↑ *Parabacteroides*  *↑ Fusicatenibacter* | 16S rRNA sequencing | Gut microbiome  Fecal sample | IADPSG criteria | - Included: Shanghai residents who had a typical diet for the Songjiang District, did not have diabetes before pregnanc  - Excluded: multiple births, diabetes, hypertension, thyroid disease, gastrointestinal or cardiovascular disease before pregnancy, use of assisted reproductive technology, antibiotic use in the previous 2 months, active smokers | Second trimester (24–28 weeks) | Total sample n= 53  -With GDM n= 21  -Without GDM n=32 | Shanghai, China | Cohort | From September 2019 to June 2020  Published:  26 August 2021 | Su, Y. et al. [53] |

**Supplementary Table 2: The extracted data related to the oral microbiome profile in patients with GDM**

| Alterations of oral microbiota profile (phylum, order, family, genus | Method of microbial analysis | Analyzed microbiome  And  Sample source | Method of diagnosis of GDM of offspring mothers | Participants characteristics | Timing of analysis | Sample size | Country and/or ethnicity | Study type | Year | Study |
| --- | --- | --- | --- | --- | --- | --- | --- | --- | --- | --- |
| Skewed prevalence for multiple genera in either gestational diabetes or the healthy state, suggesting changes in microbial composition for the oral, vaginal, and rectal compartments in women with gestational diabetes when compared with healthy pregnancies | 16S rRNA sequencing | Oral microbiome  swab specimens of the oral cavity | - | - | Third-trimester | Total sample n= 29 | Israel | Cohort | Published:  April, 2012 | Solt, I. et al. [16] |
| -Phylum:  ↑ *Proteobacteria*  *↓ Firmicutes* | 16S rRNA gene and metagenomic sequencing | Oral microbiome  Fresh saliva samples | According to the results of OGTT | Chinese pregnant women and neonates  The vast majority of the neonatal samples were collected from caesarean delivery (76 C-sections vs 17 vaginal deliveries) | 1–2 days before delivery | Total sample n= 486  (n=140 neonates and n=346 pregnant women) with n=1062 total samples collected  n=581 maternal samples(n=175 oral, n=147 intestinal and n=259 vaginal) | Chinese women | Cohort | Published:  14 May 2018 | Wang, J. et al. [18] |
| No significant differences concerning phyla and genera between the groups | 16S rRNA sequencing | Oral microbiome  Oral secretion sample | WHO  Criteria | -Included: singleton pregnancy, live fetus  - Excluded: multiple pregnancy, fetal death, autoimmune diseases, pre-existing diabetes, uterine malformations, pregnancy resulting from in vitro fertilization, placental abruption, infections, cancer, or any other systemic disease | Third trimester of gestation (28–36 weeks) | Total sample n= 68  -With GDM n=26  -Without GDM n=42 | São Paulo, Brazil | Cross-sectional study | Between 2014 and 2016  Published:  12 November 2018 | Cortez, R. V. et al. [21] |
| ↑ tuberculosis bacilli  ↑ Black-pigmented bacteria  ↑ *Capnocytophaga*  *↑ actinomycetes*  ↓ oral *streptococci*  *↓ lactobacilli* | 16S rRNA sequencing | Oral microbiome  Supragingival plaque | Standards of Medical Care in Diabetes-2011 recommended by ADA | Women aged 14–28 weeks of pregnancy  - Excluded:  taking drugs that affect blood glucose having a history of diabetes or family history of diabetes; smoking and drinking; history of severe chronic diseases, have hyperemesis gravidarum, trophoblastic disease, preeclampsia | at 28 weeks of gestation | Total sample after exclusion n=331  -With GDM n=65  -Without GDM n=266  After matching:  n=59 matched pairs of pregnant women with and without GDM | China | Non-randomized comparative study (case-control) | From February 2018 to August 2018  -Published:  March 2019 | Yao, H. et al. [45] |
| ↓ alpha diversities  no significant differences observed in beta-diversity  -Genus:  ↑ *Leptotrichia*  *↓ Bifidobacterium* | 16S rRNA sequencing | Oral microbiome  Saliva samples | WHO criteria | Excluded if: Age <18, other pregnancy complications, multiple pregnancy, pre-pregnancy diabetes, stress and anxiety, depression, probiotics and antibiotics in last 2 weeks, GI discomfort, history of smoking | Third trimester | Total sample n=61  -With GDM n=30  -Without GDM n=31 | Zhengzhou, China | Case-control | From January to August, 2018  Published:  January 1, 2020 | Xu, Y. et al. [22] |
| -No difference in richness  -OTUs:  From phylum Proteobacteria:  ↓ *Actinobacillus paraheamolyticus*  *↓ Neisseria*  -↓ *Streptococcus*  *↑ Veillonellaceae*  *↑ Prevotella* | 16S rRNA sequencing | Oral microbiome  Saliva samples | IADPSG criteria | Included: Women with and without GDM, without antibiotic treatment within a period of 2 months before the date for the OGTT test, or any medical treatment for their GDM | Third trimester (27–33 gestational weeks) | Total samples n=212  -With GDM n=50  -Without GDM n=160  -With uncertain status n=2 | Denmark,  Danish ethnicity | Longitudinal study | Published:  31 January 2020 | Crusell, M. K. W. et al. [20] |
| -Both saliva and dental plaque samples of the GDM group were divided into different clusters from those of the group without GDM  -No significant difference in a-diversity | 16S rRNA sequencing | Oral microbiome  Saliva and dental plaque samples | IADPSG criteria | Non-vegetarian and had no history of smoking, alcohol consumption, or any other systemic, metabolic or oral diseases, especially periodontitis and dental caries | Third trimester | Total sample n= 111  - With GDM n= 44  -Without GDM n=67 | China,  Han ethnicity and permanent residents of Wenzhou | Case-Control | Published:  20 January 2021 | Li, X. Q. [49] |
| -Family:  ↑ unidentified_*Flavobacteriales*  ↑ unidentified_*Bacillales*  ↑ *Christensenellaceae*  *↑ Ruminococcaceae*  *↑ Enterobacteriaceae* | 16S rRNA sequencing | Oral microbiome  Salivary, supragingival plaque and subgingival plaque samples | IADPSG criteria | Aged from 20 to 45 years.  Without digestive system diseases, metabolic diseases, or immune system diseases and tumors; had not taken antibiotics or probiotics within the preceding 3 months | In the second trimester (20–28 weeks) | Total sample n=69  - periodontitis n = 28  -With GDM n=7  - periodontitis + GDM n = 7  - periodontitis + without GDM n = 27 | China | Cohort | Published: 09 Feb 2021 | Zhang, X. et al. [23] |

**Supplementary Table 3: The extracted data related to vaginal microbiome profile in patients with GDM**

| Alterations of vaginal microbiota profile (phylum, order, family, genus) | Method of microbial analysis | Analyzed microbiome  And  Sample source | Method of diagnosis of GDM of offspring mothers | Participants characteristics | Timing of analysis | Sample size | Country and/or ethnicity | Study type | Study year | Study |
| --- | --- | --- | --- | --- | --- | --- | --- | --- | --- | --- |
| Skewed prevalence for multiple genera in either gestational diabetes or the healthy state, suggesting changes in microbial composition for the oral, vaginal, and rectal compartments in women with gestational diabetes when compared with healthy pregnancies | 16S rRNA sequencing | Vaginal microbiome  Vaginal mucosa swabs | - | - | Third-trimester | Total sample n= 29 | Israel | Cohort | Published:  April, 2012 | Solt, I. et al. [16] |
| -No obvious variation in vaginal microbiota was shown. | 16S rRNA gene and metagenomic sequencing | Vaginal microbiome  Vaginal swabs from posterior fornix | According to the results of OGTT | Chinese pregnant women and neonates  The vast majority of the neonatal samples were collected from caesarean delivery (76 C-sections vs 17 vaginal deliveries) | 1–2 days before delivery | Total sample n= 486  (n=140 neonates and n=346 pregnant women) with n=1062 total samples collected  n=581 maternal samples(n=175 oral, n=147 intestinal and n=259 vaginal) | Chinese women | Cohort | Published:  14 May 2018 | Wang, J. et al. [18] |
| -Significantly higher frequency of vulvovaginal *candidiasis*  -*Lactobacillus* was predominant in both groups  -In GDM:  *Lactobacillus listeri, Lactobacillus amylovorus, and Lactobacillus fructivorans*  *↑ L acidophilus, L crispatus, and L inersclone*  ↓ constitutional ratio of *vaginal L inersclone*  ↑ ratio of *L jensenii* | 16S rRNA sequencing | Vaginal microbiome  Vaginal secretions from the upper third of the vagina | IADPSG criteria | Han nationality; singleton pregnancy; without sex in the past week; not taking systemic or local antibiotic; and without medical history of vaginal douching or treatment | 28 to 30 and 37 to 40 weeks of pregnancy | Total sample n=386  -With GDM n=186  -Without GDM n= 200 | Beijing, China | Prospective study | Between February and October 2015  Published: 24 Aug 2018 | Zhang, X. H. et al. [55] |
| - Phylum: (no statistical significance)  ↑ Firmicutes  ↑ Proteobacteria  - Genus: (no statistical significance)  ↑ *Staphylococcus*  *↑ Megasphaera*  *↑ Shuttleworthia*  *↑ Enterobacter*  *↑ Enterococcus*  Significant alterations:  ↑ *Bacteroides*  *↑ Veillonella*  *↑ Klebsiella*  *↑ Escherichia-Shigella*  *↑ Enterococcus*  *↑ Enterobacter* | 16S rRNA sequencing | Vaginal microbiome  Vaginal cervix Sample | WHO  Criteria | -Included: singleton pregnancy, live fetus  - Excluded: multiple pregnancy, fetal death, autoimmune diseases, pre-existing diabetes, uterine malformations, pregnancy resulting from in vitro fertilization, placental abruption, infections, cancer, or any other systemic disease | Third trimester of gestation (28–36 weeks) | Total sample n= 68  -With GDM n=26  -Without GDM n=42 | São Paulo, Brazil | Cross-sectional study | Between 2014 and 2016  Published:  12 November 2018 | Cortez, R. V. et al. [21] |
| Higher alpha diversity in comparison to non-diabetic women  ↑ *Fusobacterium*  *↑ Mobiluncus*  *↑ Prevotella*  *↑ Brevibacterium*  Family:  ↑ *Enterobacteriaceae (Campylobacter, Haemophilus)*  *↑ Aerococcaceae*  *↑ Sutterellaceae*  *↑ Lachnospiraceae* | 16S rRNA sequencing | Vaginal microbiome  Swabs of Vaginal secretions | Using the 2 h 75 g oral glucose tolerance test | Pregnant women diagnosed with short cervix (<25 mm)  -Excluded:  history of sPTB, previous surgery to the cervix, evidence of premature rupture of membranes or symptomatic uterine contractions at the time of recruitment, the presence of fetal abnormalities, vaginal symptoms consistent with infection at the time of enrolment, the presence of a cervical cerclage or pessary in place at the time of enrolment | Early third trimester (23–32 weeks’ gestation) | Total sample n=46  -With GDM n=10 | Florence, Italy  predominantly Caucasian ethnicity | Cohort | Between 2014 and 2018  -Published: 10 November 2020 | Di Paola, M. et al. [30] |

**Supplementary Table 4: The extracted data related to the gut microbiome profile in the offspring of patients with GDM**

| Alterations of gut microbiota profile (phylum, order, family, genus | Method of microbial analysis | Analyzed microbiome  And  Sample source | Method of diagnosis of GDM of offspring mothers | Participants characteristics | Timing of analysis | Sample size | Country and/or ethnicity | Study type | Study year | Study |
| --- | --- | --- | --- | --- | --- | --- | --- | --- | --- | --- |
| ↑ levels of beta-diversity in infants age 1 month and 6 months, but by 4 years of age, children had levels of beta-diversity similar to mothers at T1 (at 13.84 ± 0.16 weeks of gestation) | 16S rRNA sequencing | Gut microbiome  Fecal sample | - | Women who were previously recruited for a prospective, randomized mother-infant nutrition study in Finland and their offspring | 1 month of age, 6 months and 4 years of age | Total sample n= 91  -With GDM n=15  -Without GDM n=76 | Finland | Cohort | Published:  August 2, 2012 | Koren, O. et al. [17] |
| -DM and GDM groups were each significantly different from the no-diabetes group  -no significant difference was observed between the DM and GDM groups  -Phylum:  In comparison with the adult stool samples:  ↑ Proteobacteria  ↓ Bacteroidetes  ↓Firmicutes  In DM group:  ↑ Bacteroidetes  ↓ Proteobacteria  -OTUs:  Family:  ↑ *Lachnospiraceae*  Genus:  ↑ *Parabacteriodes* (similar to adults with DM) | 16S rRNA sequencing | Meconium | According to the results of OGTT | -Excluded: any antibiotic treatment during pregnancy (except women who eventually underwent C-section and received an immediate dose of Kefzol as a standard of care), or obstetric risks | Between 2 hours and 48 hours after birth | Total newborns N=23  -n=4 mothers had pre-gestational type 2 diabetes mellitus (DM)  -n= 1 mother with dizygotic twins  -n=5 with GDM  -n= 13 had no diabetes | New York City, United Stated of America | Case-control | Published:  November 6, 2013 | Hu, J. et al. [33] |
| Correlated with GDM across multiple sample types:  *Blautia*  *Coprococcus*  *Roseburia*  *Sutterella* | 16S rRNA gene and metagenomic sequencing | Multiple body sites, including meconium | According to the results of OGTT | Chinese pregnant women and neonates  The vast majority of the neonatal samples were collected from caesarean delivery (76 C-sections vs 17 vaginal deliveries) | Within 24hours as soon as the newborns passed their first intestinal discharge | Total sample n= 486  (n=140 neonates and n=346 pregnant women) with n=1062 total samples collected  n=233 samples failed to generate data  -eligible n= 248 neonatal (64 amniotic fluid, 20 oral, 81 pharyngeal and 83 meconium) | Chinese women | Cohort | Published:  14 May 2018 | Wang, J. et al. [18] |
| In comparsion to control group:  lower alpha-diversity  -Phylum:  ↑ Proteobacteria  ↑ Actinobacteria  ↓ Bacteroidetes (in GDM_A1)  -Genus:  ↓ *Prevotella* in GDM_A1  ↓ *Lactobacillus* in GDM_A1, and ↑ in GDM_A2 without statistical significance | 16S rRNA sequencing | Meconium (Faeces from diapers) | IADPSG criteria | Full-term and C-sectioned newborns,  -Excluded: any infection occurring during pregnancy, use of any type of antibiotic during pregnancy excluding the antibiotic administered before caesarean section as standard obstetric care; pregnant women with any other complications, newborns administrated any type of antibiotic after birth, any obstetric risks | within 24 hours after birth | Total sample n=34  -Offspring of women without GDM n=14  -Offspring of women with GDM n=20  Classified into:  - GDM_A1 n=15 who were treated by diet and exercise  - GDM_A2 n=5 who were treated by insulin in addition to diet and exercise | West China | Longitudinal/prospective | From August 31, 2016 to January 10, 2017  Published:  October 17, 2018 | Su, M. et al. [56] |
| Lower α-diversity than the corresponding mothers  In comparison to mothers gut microbiota (both with & without GDM):  -Phylum:  ↑ Actinobacteria and ↑Proteobacteria  -Genus:  ↑ *Bifidobacterium*  *↑ Streptococcus*  *↑ Escherichia*  *↑ Staphylococcus*  *↑ Enterococcaceae*  In comparison to offspring of women without GDM:  -Phylum  ↑ Actinobacteria  ↑ Bacteroidetes  -Genus:  ↑ *Escherichia*  ↑ *Parabacteroides* | 16S rRNA sequencing | Gut microbiome    Fecal samples | International guidelines | Infants of women diagnosed with GDM  Who did not have twin pregnancy, any pathological conditions before or during pregnancy, no compliance to the study protocol, on prebiotics/probiotics, antibiotics or any drug during pregnancy | During the first week of life  between the 3rd and the 5th day of life, after meconium expulsion | n= 50 mothers had GDM  n=41 mothers out of 50 participated in the observational study  n=12 stool samples out of 41 were inappropriate    n= 29 infants remained of mothers with GDM  n=19 infants of women without GDM | Turin, Italy  European origin with both parents born in Europe | Cohort | Published:  December 16, 2019 | Ponzo, V. et al. [31] |
| Fewer OTUs compared with neonates born to mothers without GDM  In neonates:  From Phylum Firmicutes:  -Genus: ↑ *Isobaculum*  ↑ the parent family *Carnobacteriaceae*  *↑ Turicibacter*  *↓ Veillonella*  *↓ Megasphaera*  *↓ Subdoligranulum*  *↓ Ethanoligenes*  From Phylum Bacteroidetes:  -↓ Genus *Prevotella*  ↓ parent family *Prevotellaceae*  From Phylum Actinobacteria:  -Genus:  ↓ *Rothia*  ↓ parent family *Micrococcaceae* | 16S rRNA gene sequencing | Fecal samples  (2 days after the offspring had passed meconium) | IADPSG criteria | Included: Women with and without GDM, without antibiotic treatment within a period of 2 months before the date for the OGTT test, or any medical treatment for their GDM | During the first week of life (range 2–7 days of life, n = 123) and  and again at least 6 months after birth [8.8 months on average (8–9, (n = 124) | Total sample n=125  -n = 43  children of mothers with GDM  -n = 82 children of mothers without GDM | Hvidovre and Herlev, Denmark | Cohort | Published:  23 October 2020 | Crusell, M. K. W. et al. [25] |
| ↓ *Lactobacillus*  *↓ Flavonifractor*  *↓ Erysipelotrichaceae*  ↓ unspecified families in *Gammaproteobacteria*  *↑ Phascolarctobacterium* | 16S rRNA sequencing | Stool sample | Carpenter and Coustan criteria | -Included: neonates born to normal-weight or overweight/obese mothers with and without GDM.  Vaginal delivery or cesarean delivery after trial of labor (ensuring exposure to the vaginal canal and associated microbiota), no antibiotics or probiotics except in the immediate peripartum period, predominate breastfeeding in the first 2 weeks of life.  Women with GDM were included only if managed by diet alone, without the use of medications (such as insulin or oral agents) | 2-week-old (postpartum) collected within 24 h of visit | Total sample n= 46 pairs of mothers-infants:  N=4 delivered via cesarean delivery after a trial of labor  N=4 infants had formula supplement  N=1 one mother had taken probiotics consistently for greater than 2 years  -N=5 were given penicillin at delivery for Group B Streptococcus -n=1 received doxycycline antibiotics during the 2-week postpartum period | Denver, Colorado metropolitan area. | Cohort | Between 2012 and 2017  Published:  27 November 2020 | Soderborg, T. K. et al. [32] |
| In comparison to control group:  -Phylum:  ↑ Firmicutes  ↓ Proteobacteria  -Family:  ↑ *Streptococcaceae*  -Genus:  ↑ Rothia  ↑ Clostridium sensustricto | 16S rRNA gene sequencing | Meconium | WHO criteria | -Excluded:  Pre-existing diabetes, pre-existing metabolic diseases, antibiotics usage within 3 months, alcohol or substance abuse, and chronic diseases requiring medication | Within the first few hours of birth at the labor ward | Total sample n=455 mothers and their neonates  -n=37 samples were removed due to the low reads number  -N=418 were retained:  -n=147 women with GDM  -n= 271 normal pregnant women | Nanjing, Jiangsu Province, China) | Cross-sectional | Published:  27 May 2021 | Chen, T. et al. [42] |
| No alterations were observed in meconium | 16S rRNA sequencing | Gut microbiome  Meconium samples | NDDG criteria | - Included: Singleton pregnant women  -Excluded: medical diseases prior to pregnancy, multifetal gestation, fetal or neonatal anomaly, receiving corticosteroids, beta-blockers, and antibiotics at the time of GDM diagnosis, incomplete data or unknown pregnancy outcomes | Within 24 and 48 h after delivery | Total sample n=88 and their 88 offspring  -With GDM n=49  -Without GDM n= 39 | Chiang Mai, Thailand | Longitudinal prospective | August 2019 to February 2020  Published:  8 August 2021 | Sililas, P. et al. [24] |

**Supplementary Table 5: The extracted data related to the oral microbiome profile in the offspring of patients with GDM**

| Alterations of oral microbiota profile (phylum, order, family, genus) | Method of microbial analysis | Analyzed microbiome  And  Sample source | Method of diagnosis of GDM of offspring mothers | Participants characteristics | Timing of analysis | Sample size | Country and/or ethnicity | Study type | Study year | Study |
| --- | --- | --- | --- | --- | --- | --- | --- | --- | --- | --- |
| Consistency of microbial variation across mothers with GDM and their neonates in bacterial abundance | 16S rRNA gene and metagenomic sequencing | Oral microbiome  multiple body sites, including saliva and pharyngeal aspirates | According to the results of OGTT | Chinese pregnant women and neonates  The vast majority of the neonatal samples were collected from caesarean delivery (76 C-sections vs 17 vaginal deliveries) | Within 24hours as soon as the newborns passed their first intestinal discharge | Total sample n= 486  (n=140 neonates and n=346 pregnant women) with n=1062 total samples collected  n=233 samples failed to generate data  -eligible n= 248 neonatal (64 amniotic fluid, 20 oral, 81 pharyngeal and 83 meconium) | Chinese women | Cohort | Published:  14 May 2018 | Wang, J. et al. [18] |
| -Higher alpha-diversity  -Phylum:  ↑ Bacteroidetes  -Class:  ↑ Bacteroidia  ↑ Clostridia  -Genus:  ↑ Alistpes  ↑ Streptococcus  ↑ Faecalibacterium | 16S rRNA sequencing | Oral microbiome  Oral swabs | IADPSG criteria | Infants born with gestational age from 37 to 42 weeks, birth weight >2,500 g, without any significant congenital anomalies, neurological dysfunction, fetal chromosomal abnormalities, metabolic diseases or need resuscitation after birth | 1 min after birth | Total sample n= 20  -Offspring of women with GDM n=9  -Offspring of women without GDM n=11 | Shenzhen, China | Prospective randomized pilot study | In 2016  Published: 10 December 2019 | He, Z. J. et al. [57] |
| -Genus:  ↑ Alistipes  ↑ Streptococcus  ↑ Faecalibacterium  ↑ Prevotella  ↑ Bacteroidetes  ↑ Bifidobacterium  ↑ Corynebacterium  ↑ Ureaplasma  ↑ Weissella | 16S rRNA sequencing | Oral microbiome  Oral swabs | IADPSG criteria | Vaginally delivered neonates, with gestational age 37–42 weeks, infants with birth weight > 2500 g, and infants without any significant congenital and fetal chromosomal abnormalities. | 1 min after birth | Total sample n= 155  -Offspring of women with GDM n=75  -Offspring of women without GDM n=80 | India | Case Control | Published:  23 December 2020 | Singh, P. et al. [37] |

**Supplementary Table 6: The extracted data related to placental microbiome in the offspring of patients with GDM**

| Alterations of placental microbiota profile (phylum, order, family, genus) | Method of microbial analysis | Analyzed microbiome  And  Sample source | Method of diagnosis of GDM of offspring mothers | Participants characteristics | Timing of analysis | Sample size | Country and/or ethnicity | Study type | Study year | Study |
| --- | --- | --- | --- | --- | --- | --- | --- | --- | --- | --- |
| -From phylum Actinobacteria:  Order: ↑ *Coriobacteriales*  Family: ↑ *Coriobacteriaceae*  -From phylum Bacteroidetes:  Genus: ↑ *Parabacteroides*  -From phylum Firmicutes:  Family: ↑ *Lachnospiraceae*  -From phylum Proteobacteria:  Family: ↑ *Bradyrhizobiaceae*  *↑ Escherichia* | 16S rRNA sequencing | Placental microbiome  Placental samples | A 50-g, 1-h and 100-g, 3-h glucose tolerance test | Infants born at term (37-42 weeks) to women with and without GDM | After childbirth in delivery room | Total sample n=22  -With GDM n=11  -Without GDM n=11 | Girona, Spain  Caucasian women | Cross- sectional study | Published:  4 August 2016 | Bassols, J. et al. [39] |
| -Phylum:  ↑ Proteobacteria  ↓ Bacteroidetes  ↓ Actinobacteria  ↓ Firmicutes  -Genus:  ↓ *Bacteroidales_S24-7_group_norank*  *↓ Lactobacillus*  *↓ Rhodocyclaceae_norank Brevibacillus ↓ Armatimonadetes_norank*  *↓ Candidatus_Brocadia*  *↓ PHOSHE36_norank*  *↓ Sulfuricella*  *↓ Xanthomonadales_uncultured*  *↓ Geothrix*  *↓ TRA3-20_norank*  *↓ Cyanobacteria_norank*  *↓ Dyella*  ↑ *Alcaligenaceae_unclassified*  *↑ Thauera* | 16S rRNA sequencing | Placental microbiome  Placental samples | IADPSG criteria | Excluded:  Women with any severe illnesses before pregnancy, treatment with any medications during pregnancy, multiparous pregnancy and fetuses with genetic or congenital malformations, asphyxia at birth, neurological dysfunction or any other diseases, the presence of any antepartum infections and antibiotic treatment during pregnancy. | Immediately after delivery | Total sample n= 20  -With GDM n=10  -Without GDM n=10 | Beijing, China  Chinese Han ethnicity | Pilot-study | Published: 06 September 2017 | Zheng, J. et al. [59] |
| -In the pilot study: samples contained levels of bacterial 16S rRNA gene that were below the limit of accurate detection in the assay, no statistical difference was observed between the total placental samples and blanks  -In the expanded cohort study:  could not distinguish samples from GDM, normal BMI, and obese patients due to very low reads | 16S rRNA sequencing | Placental microbiome  Placental parenchyma | ACOG criteria | -Excluded: gestational age < 37 0/7 weeks or > 41 6/7 weeks, late prenatal care or inaccurate gestational dating, onset of labor, rupture of membranes, antibiotic treatment in the third trimester, incomplete medical records, pregestational diabetes, or pre-pregnancy BMI > 25 but < 30 (overweight but not obese mothers) | Immediately after cesarean section delivery | Total sample in the pilot study n=17  -With GDM n=6  - normal BMI n=5  - obese n=6  In the expanded cohort:  n= 10 per group | Lansing, Michigan | Case- Control Pilot study expanded to cohort study | Published:  11 June 2020 | Olomu, I. N. et al. [47] |
| - More operational taxonomic units in GDM group  -Genus:  ↑ *Ruminococcus*  *↑ Coprococcus*  *↑ Paraprevotella*  *↑ Lactobacillus*  *↓ Veillonella* | 16S rRNA sequencing | Placental microbiome  Placental samples | - | -Excluded:  vaginal delivery; preterm birth; preexisting diabetes before pregnancy, or hypertension/preeclampsia, clinical chorioamnionitis, or other severe pregnancy complication, women having any clinical infection or who were treated with any antibiotics during pregnancy, birth defects, any antepartum infection during pregnancy | Following cesarean delivery | Total sample n= 15  -With GDM n=8  -Without GDM n=7 | Shanghai, China | Cross- Sectionnal study | Published:  21 October 2020 | Tang, N. et al. [58] |

**Supplementary Table 7: The extracted data related to postpartum alterations in the microbiome of patients with GDM**

| Alterations of gut microbiota profile postpartum (phylum, order, family, genus) | Method of microbial analysis | Analyzed microbiome  And  Sample source | Method of diagnosis of GDM of offspring mothers | Participants characteristics | Timing of analysis | Sample size | Country and/or ethnicity | Study type | Study year | Study |
| --- | --- | --- | --- | --- | --- | --- | --- | --- | --- | --- |
| Persisted high levels of between-individual variation in community composition observed in T3  -Genus:  ↑ *Streptococcus* | 16S rRNA sequencing | Gut microbiome  Fecal sample | - | Women who were previously recruited for a prospective, randomized mother-infant nutrition study in Finland | 1 month postpartum | Total sample n= 91  -With GDM n=15  -Without GDM n=76 | Finland | Cohort | Published:  August 2, 2012 | Koren, O. et al. [17] |
| -Alpha diversity was similar between the two groups and no significant differences on lower taxonomic levels were observed  -Phylum:  ↓ Firmicutes  -Family:  ↑ *Prevotellaceae* | 16S rRNA sequencing | Gut microbiome postpartum  Fecal sample | Oral glucose tolerance test (OGTT) | Women with GDM during their last pregnancy and women following a normoglycemic pregnancy | 3–16 months after delivery | Total sample n= 77  -With previous GDM (pGDM) n=42  -Control n=35 | Munich, Germany | Cross-sectional  Prospective observational study | between November 2011 and December 2013  Published:  17 August 2015 | Fugmann, M. et al. [36] |
| - No difference in alpha diversity  -significant reduction in the number of observed OTUs  - ↑ Seven OTUs in women with previous GDM including two *Faecalibacterium* OTUs  -↓ Six OTUs:  *Faecalibacterium*  *Bacteroides*  *Isobaculum* | 16S rRNA sequencing. | Gut microbiome  Fecal sample | IADPSG criteria | - Included: singleton pregnant, Danish white origin , without diagnosed pre-eclampsia at the time of inclusion, multipara women with a previous normoglycaemic pregnancy  - Excluded:  antibiotics within a period of 2 months before the first visit | 8 months postpartum | Total sample of re-examined women postpartum n= 125  -With GDM n=43  -Without GDM n= 82 | Denmark  Danish white origin | Cohort | January 2014 to February 2015  Published:  15 May 2018 | Crusell, M.K.W. et al. [19] |
| ↓ Richness from pregnancy to postpartum  -OTUs:  ↑ *Bacteriodales*  *↑ Treponema*  *↓ Leptotrichia*  ↓ *Streptococcus*  *↓ Neisseria*  ↓ unclassified *Bacteria*  ↓ *Weeksellaceae*  *↓ Atopobium* | 16S rRNA sequencing | Oral microbiome  Saliva samples | IADPSG criteria | Included: Women with and without GDM, without antibiotic treatment within a period of 2 months before the date for the OGTT test, or any medical treatment for their GDM | 9 months postpartum | Total samples n=125  -With GDM n=43  -Without GDM n=81 | Denmark,  Danish ethnicity | Longitudinal study | Published:  31 January 2020 | Crusell, M. K. W. et al. [25] |
| In GI group:  -Phylum:  ↑ Bacteroidetes  ↓ Firmicutes  -Genus:  ↑ *Prevotella_9*  *↑ Bacteroides* | 16S rRNA sequencing | Gut microbiome postpartum  Fecal sample | CPG | Excluded:  post-GDM women on regular treatment, including diabetic therapy, anti-inflammatory drugs, laxatives, traditional medicine, or antibiotics/probiotics within 3 months prior to the present study | -6–8 weeks postpartum  -between 3 and 6 months postpartum) | Total sample with previous GDM n=35  After exclusion: n=24  Divided into 2 groups:  - With postpartum glucose intolerance (GI) n= 14  -With normal glucose tolerance (NGT) n= 10 | Malaysia  Malay ethnicity | Cross-sectional | From March 2018 to February 2020  Published:  25 June 2021 | Hasain, Z. et al. [38] |

Abbreviations:

American College of Obstetricians and Gynecologists (ACOG), American Diabetes Association (ADA), Body mass index (BMI), Cesarean section (C-section), Gestational Diabetes Mellitus (GDM), International Association of Diabetes and Pregnancy Study Groups (IADPSG), Malaysian Clinical Practice Guidelines (CPG), National Diabetes Data Group (NDDG), Oral glucose tolerance test (OGTT), 16S ribosomal ribonucleic acid (16S rRNA), World Health Organization (WHO)

**Supplementary Table 14: Quality assessment of the case-control studies. = High-quality studies.**

| Study | Selection | Comparability | Exposure/ Outcome | Total |
| --- | --- | --- | --- | --- |
| Yu H. et al. [41] | 3 | 0 | 2 | 5 |
| Wu, Y. et al. [44] | 3 | 0 | 2 | 5 |
| Xu, Y. et al. [22] | 3 | 2 | 2 | 7 |
| Hou, M. et al. [51] | 3 | 0 | 2 | 5 |
| Ma, S. J. et al. [63] | 3 | 2 | 2 | 7 |
| Zheng, W. et al. [43] | 3 | 2 | 2 | 7 |
| Mokkala, K. et al. [26] | 3 | 2 | 2 | 7 |
| Hu, P. et al. [64] | 3 | 2 | 2 | 7 |
| Wang, X. et al. [62] | 3 | 2 | 2 | 7 |
| Chen, T. Et al. [52] | 3 | 2 | 2 | 7 |
| Li, X. Q. [49] | 3 | 0 | 2 | 5 |
| Hu, J. et al. [33] | 3 | 0 | 2 | 5 |
| Singh, P. et al. [37] | 3 | 0 | 2 | 5 |
| Yao, H. et al. [45] | 3 | 2 | 2 | 7 |

**Supplementary Table 15: Quality assessment of the cross-sectional studies. = High-quality studies.**

| Study | Selection | Comparability | Exposure/ Outcome | Total |
| --- | --- | --- | --- | --- |
| Cortez, R. V. et al. [21] | 3 | 0 | 2 | 5 |
| Ye, G. et al. [65] | 3 | 0 | 2 | 5 |
| Cui, M. J. et al. [46] | 3 | 0 | 2 | 5 |
| Festa, C. et al. [28] | 2 | 2 | 2 | 6 |
| Li, G. et al. [50] | 3 | 0 | 2 | 5 |
| Chen, T. et al. [42] | 3 | 2 | 2 | 7 |
| Bassols, J. et al. [39] | 3 | 2 | 2 | 7 |
| Tang, N. et al. [58] | 3 | 0 | 2 | 5 |
| Fugmann, M. et al. [36] | 3 | 0 | 2 | 5 |
| Hasain, Z. et al. [38] | 3 | 0 | 2 | 5 |
| He, Z. J. et al. [57] | 2 | 0 | 2 | 4 |
| Zheng, J. et al. [59] | 2 | 2 | 2 | 6 |

**Supplementary Table 16: Quality assessment of the cohort studies. = High-quality studies.**

| Study | Selection | Comparability | Exposure/ Outcome | Total |
| --- | --- | --- | --- | --- |
| Solt, I. et al. [16] | 0 | 0 | 0 | 0 |
| Koren, O. et al [17] | 4 | 0 | 3 | 7 |
| Mokkala, K. et al. [27] | 4 | 2 | 3 | 9 |
| Kuanget, Y. S. et al. [40] | 4 | 0 | 3 | 7 |
| Wang, J. et al. [18] | 4 | 0 | 3 | 7 |
| Crusell, M.K.W. et al. [19] | 4 | 2 | 3 | 9 |
| Ferrocino, I. et al. [29] | 4 | 2 | 3 | 9 |
| Liu, H. et al. [54] | 4 | 1 | 3 | 8 |
| Wu, Y. et al. [44] | 4 | 0 | 3 | 7 |
| Dong, L. N. et al. [60] | 4 | 0 | 3 | 7 |
| Zhang, X. et al. [23] | 4 | 0 | 3 | 7 |
| Chen, F. et al. [61] | 4 | 0 | 3 | 7 |
| Mullins, T. P. et al. [35] | 4 | 2 | 3 | 9 |
| Wei, J. et al. [48] | 4 | 2 | 3 | 9 |
| Su, Y. et al. [53] | 4 | 0 | 3 | 7 |
| Di Paola, M. et al. [30] | 4 | 2 | 3 | 9 |
| Ponzo, V. et al. [31] | 4 | 2 | 3 | 9 |
| Crusell, M. K. W. et al. [25] | 4 | 2 | 3 | 9 |
| Soderborg, T. K. et al. [32] | 4 | 2 | 3 | 9 |
| Olomu, I. N. et al. [47] | 4 | 0 | 3 | 7 |
| Sililas, P. et al. [24] | 4 | 0 | 3 | 7 |
| Crusell, M. K. W. et al. [20] | 4 | 2 | 3 | 9 |
| Su, M. et al. [56] | 4 | 0 | 3 | 7 |
